# Tumor-associated macrophage-derived IL-6 and IL-8 enhance invasive activity of LoVo cells induced by PRL-3 in a KCNN4 channel-dependent manner

**DOI:** 10.1186/1471-2407-14-330

**Published:** 2014-05-10

**Authors:** Heyang Xu, Wei Lai, Yang Zhang, Lu Liu, Xingxi Luo, Yujie Zeng, Heng Wu, Qiusheng Lan, Zhonghua Chu

**Affiliations:** 1 Department of Gastroenteropancreatic Surgery, Sun Yat-sen Memorial Hospital, Sun Yat-sen University, Guangzhou 510120, P.R. China

**Keywords:** Tumor-associated macrophage, PRL-3, IL-6, IL-8, KCNN4, CRC

## Abstract

**Background:**

Tumor-associated macrophages (TAMs) are known to promote cancer progression and metastasis through the release of a variety of cytokines. Phosphatase of regenerating liver (PRL-3) has been considered as a marker of colorectal cancer (CRC) liver metastasis. Our previous research suggests that PRL-3 can enhance the metastasis of CRC through the up-regulation of intermediate-conductance Ca^2+^-activated K^+^ (KCNN4) channel, which is dependent on the autocrine secretion of tumor necrosis factor-alpha (TNF-α). However, whether TAMs participate in the progression and metastasis of CRC induced by PRL-3 remains unknown.

**Methods:**

We used flow cytometry, coculture, western blotting, invasion assays, real-time quantitative PCR, chromatin immunoprecipitation, luciferase reporter assays, and immunofluorescence staining to determine the effect of TAMs on the ability of PRL-3 to promote invasiveness of CRC cells.

**Results:**

In this study, we found that TAMs facilitated the metastasis of CRC induced by PRL-3. When TAMs were cocultured with CRC cells, the expression of KCNN4 was increased in TAMs and the invasion of CRC cells was enhanced. Furthermore, cytokines that were secreted by TAMs, such as IL-6 and IL-8, were also significantly increased. This response was attenuated by treating TAMs with the KCNN4 channel-specific inhibitor, 1-[(2-chlorophenyl) diphenylmethyl]-1H-pyrazole (TRAM-34), which suggested that KCNN4 channels may be involved in inducing the secretion of IL-6 and IL-8 by TAMs and improving CRC cell invasiveness. Moreover, the expression of KCNN4 channels in TAMs was regulated through the NF-κB signal pathway, which is activated by TNF-α from CRC cells. Immunofluorescence analysis of colorectal specimens indicated that IL-6 and IL-8 double positive cells in the stroma showed positive staining for the TAM marker CD68, suggesting that TAMs produce IL-6 and IL-8. Increased numbers of these cells correlated with higher clinical stage.

**Conclusions:**

Our findings suggested that TAMs participate in the metastasis of CRC induced by PRL-3 through the TNF-α mediated secretion of IL-6 and IL-8 in a paracrine manner.

## Background

Immune cells infiltrate all neoplastic lesions, and together, the immune cells and tumor cells constitute the tumor microenvironment. Such immune cells were previously thought to function in the defense response against the tumor [1]. However, recently, increasing evidence indicates that tumor-associated inflammatory cells may enhance tumor progression, and among these cells, macrophages play the most important role [2]. Macrophages can alter their profiles such that they become M1 or M2 macrophages according to the tumor microenvironment. It has been shown that M2 macrophages are a type of tumor-associated macrophage (TAM) [3]. TAMs can promote tumor cell growth and metastasis, and recent research has indicated that TAMs can stimulate colorectal cancer cell invasion by upregulating matrix metalloproteinase (MMP) expression and activating epidermal growth factor receptor (EGFR) [4].

Phosphatase of regenerating liver (PRL-3), a protein tyrosine phosphatase, has been demonstrated to play an important role in colorectal cancer progression and metastasis [5]. PRL-3 is significantly elevated (>90%) in metastases and moderately elevated (25–45%) in primary colorectal cancer tumor. Moreover, the expression of PRL-3 in primary tumors indicated their tendency toward liver metastasis [6]. Our previous studies have demonstrated that PRL-3 can promote the proliferation and metastasis of tumor cells through the autocrine secretion of tumor necrosis factor-alpha (TNF-α), which induces intermediate-conductance Ca^2+^-activated K^+^ (KCNN4) channel expression by activating the NF-κB signaling pathway [7]. Previous studies also revealed that TNF-α contributed to tumor progression in a paracrine manner. Because TNF-α secreted from tumor cells and/or macrophages can affect the phenotype of these cells in a paracrine and/or autocrine manner, we hypothesize that colorectal cancer cells may interact with TAMs in the microenvironment and alter the cytokine profile of TAMs to promote tumor progression and metastasis through TNF-α, the secretion of which is stimulated by PRL-3 in a paracrine manner.

Interleukin-6 (IL-6) is a potent pleiotropic cytokine that is predominantly produced by monocytes and macrophages during chronic inflammation [8]. IL-6 has been shown to be involved in tumor progression and metastasis through STAT3 signaling pathways [9]. Moreover, the level of IL-6 is positively correlated with poor prognosis in different cancers. In addition to IL-6, interleukin-8 (IL-8) is also a known proinflammatory cytokine [10]. Extensive studies have demonstrated that the levels of IL-8 and its receptor CXCR2 are significantly increased in colorectal cancer (CRC) cells, and that these proteins play an important role in tumor development [11]. Similarly, the expression of IL-8 is correlated with tumor size and tumor stage. However, the question of whether IL-6 and IL-8 are involved in the metastasis of CRC induced by PRL-3 remains unclear.

In this study, we aimed to investigate whether TAMs participate in the metastasis of CRC, which is induced by PRL-3 in the tumor microenvironment. Our study revealed that PRL-3 could induce the expression of IL-6 and IL-8 secreted by TAMs through TNF-α released by CRC cells in a paracrine manner. Further study also revealed that such regulation could be inhibited by blocking KCNN4 channels expressed by TAMs.

## Methods

### Reagents and antibodies

G418, PMA, and Lipofectamine2000 were purchased from Sigma (St Louis, Missouri, USA). Fetal bovine serum (FBS) was purchased from BioInd (Kibbutz Beit Haemek, Israel). RPMI was purchased from Invitrogen (Carlsbad, CA, USA). Trizol and Prime Script RT were purchased from Takara (Dalian, China). Matrigel matrix was purchased from BD Biosciences (Biosciences, Bedford, MD,USA). siRNA was purchased from GenePharma (Shanghai, China). Antibodies against GAPDH (cat: ab8245), KCNN4 (cat: ab83740), p50 (cat: ab7971), and p65 (cat: ab7970) were purchased from Abcam(Cambridge, MA, USA). Antibodies against CD68 (cat: sc-393951) and CD206 (cat: sc-376108) were purchased from Santa Cruz (Santa Cruz, CA, USA). Antibodies against IL-6 (cat: 1457–1) and IL-8 (cat: 3518–1) were purchased from Epitomics (Burlingame, CA, USA).

### Cell cultures and treatment

LoVo cells were purchased from the Shanghai Cell Bank of the Chinese Academy of Sciences and then transfected with PAcGFP-PRL-3 (LoVo-P) or PAcGFP (LoVo-C) using Lipofectamine2000. Stable clones were selected by culturing with 600 ug/ml G418 for 3 weeks. THP-1 cells were obtained from Shanghai Cell Bank of the Chinese Academy of Sciences. Both LoVo and THP-1 cells were cultured in RPMI 1640, supplemented with 10% fetal bovine serum (FBS), 100 mg/ml penicillin, and 100 mg/ml streptomycin. The cells were incubated at 37°C, 5% CO_2_ in a humidified atmosphere. M2-polarized THP-1 cells were generated by phorbol myristate acetate (PMA) treatment (320 nM/10^6^ cells) for 6 h followed by incubation with IL-4 (20 ng/ml) for 18 h.

### Samples and patients

CRC samples were obtained from 71 patients, who were admitted to the Department of Gastroenteropancreatic Surgery of Sun Yat-sen Memorial Hospital, Sun Yat-sen University from 2005 to 2007. Surgically resected specimens were collected immediately after tumor removal. All samples were collected with informed consent according to the Internal Review and the Ethics Boards of the Sun-Yat-Sen Memorial Hospital of Sun-Yat-Sen University. The protocol was approved by the Ethics Committee of Sun Yat-Sen Memorial Hospital.

### Flow cytometry

Cells were washed in phosphate buffered saline (PBS), resuspended and then stained with murine anti-human CD68 or CD206 for 30 min, then washed and incubated with PE-conjugated goat anti-mouse secondary antibody. Cells were analyzed by flow cytometry (BD FACS Cabiler).

### M2 macrophage and LoVo cell coculture

1 × 10^6^ M2 macrophage cells were seeded into six-well plates, while LoVo cells were cocultured with M2 macrophages in upper transwell inserts. After coculturing, LoVo and M2 macrophage cells were washed and used for later experiments.

### Enzyme linked immunosorbent assay (ELISA)

TNF-α was assayed in the culture supernatant of LoVo-P or LoVo-C cells using the Quantikine Kit (R&D Systems, Minneapolis, MN) according to the manufacturer’s protocol.

### Western blot assay

Cells were lysed on ice with RIPA buffer containing 1% PMSF. Sample protein concentration was determined by the Bradford assay. Denatured proteins were separated by 10% or 12% sodium dodecyl sulfate-polyacrylamide gel electrophoresis, transferred to polyvinylidene fluoride membranes and then blocked in 5% non-fat milk. Membranes were washed 3 times with Tris-buffered saline + 0.1% Tween-20 (TBST), incubated with relevant primary antibodies overnight at 4°C, washed and incubated for 1 h at room temperature with horseradish peroxidase-conjugated secondary antibodies. Labeled proteins were visualized by chemiluminescence.

### siRNA mediated gene suppression

siRNAs targeting human p50 cDNA and p65 cDNA were purchased from Shanghai GenePharma. The siRNA sequences, and their non-inhibitory controls, were as follows: p50: 5′-CGCCAUCUAUGACAGUAAATT-3′; control: 5′-UUCUCCGAACGUGUCACGUTT-3′; p65: 5′-GGACAUAUGAGACCUUCAATT-3′; control: 5′-ACGUGACACGUUCGGAGAATT-3′; *KCNN4* sense: 5′-GCCGUGCGUGCAGGAUUUA-3′; anti-sense: 5′-UAAAUCCUGCACGCACGGC-3′; Lipofectamine 2000 was used to transfect siRNA into M2 macrophage according to the manufacturer’s protocol.

### Cell invasion assays

Transwell inserts were used to perform cell invasion assays. After coating the upper chamber with Matrigel, 1 × 10^5^ cells in 0.2 ml serum-free RPMI 1640 medium were added. The lower chamber contained 0.8 ml medium with 10% FBS. After incubating at 37°C, 5% CO_2_ for 24 h, cells that had migrated to the lower chamber were fixed with 4% paraformaldehyde, and stained with 0.1% crystal violet in methanol, then counted under a microscope.

### mRNA extraction and real time quantitative RT-PCR

Total RNA was extracted using Trizol, and reverse transcribed using PrimeScript RT from 500 ng RNA according to the manufacturer’s protocol. Quantitative real-time RT-PCR was performed using the LightCycler 480 (Roche, Basel, Switzerland) and SYBR Assays (Takara, Dalian, China). Primers were designed to detect *CCL2, CXCL12, CCL17, CCL18, CCL22, EGF, IL-1, IL-6, IL-8, IL-10, VEGFA, TGF-β* and *GAPDH*. Oligonucleotide sequences of qRT-PCR primers are shown in Table 1. Each sample contained 1× SYBR Premix Ex TaqTM, 0.2 μM of each forward and reverse primers and 500 ng template cDNA in a final volume of 20 μl. Cycling parameters were set as follows: denaturation at 95°C for 30 s, followed by 40 amplification cycles (95°C for 5 s and 60°C for 20 s). For relative quantification, 2^-ΔΔCt^ was used to calculate the fold change in gene expression. All of the experiments were performed in triplicate.

**Table 1:** Oligonucleotide sequence of qRT-PCR primers

**Gene**	**Forward primer**	**Reverse primer**	**Amplicon(bp)**
CXCL12	5′-CCCGAAGCTAAAGTGGATTC-3′	5′-TTCAGAGCTGGGCTCCTACT-3′	112
CCL18	5′-CTCTGCTGCCTCGTCTATACCT-3′	5′-CTTGGTTAGGAGGATGACACCT-3′	108
CCL17	5′-AGGGACCTGCACACAGAGAC-3′	5′-CTCGAGCTGCGTGGATGTGC-3′	133
CCL22	5′-ATGGCTCGCCTACAGACTGCACTC-3′	5′-CACGGCAGCAGACGCTGTCTTCCA-3′	114
IL-6	5′-AATAACCACCCCTGACCCAAC-3′	5′-ACATTTGCCGAAGAGCCCT-3′	149
IL10	5′-AACAAGAGCAAGGCCGTGG-3′	5′-GAAGATGTCAAACTCACTCATGGC-3′	93
IL-1	5′-TCTGTTCTTGGGAATCCATGG-3′	5′-TCAGTGATGTTAACTGCCTCCAG-3′	96
IL-8	5′-AAACCACCGGAAGGAACCAT-3′	5′-CCTTCACACAGAGCTGCAGAAA-3′	101
CCL2	5′-AAGATCTCAGTGCAGAGGCTCG-3′	5′-CACAGATCTCCTTGGCCACAA-3′	103
EGF	5′-CTTGTCATGCTGCTCCTCCTG-3′	5′-TGCGACTCCTCACATCTCTGC-3′	118
VEGFA	5′-ATGACGAGGGCCTGGAGTGTG-3′	5′-CCTATGTGCTGGCCTTGGTGAG-3′	91
TGF-β	5′-AAGGACCTCGGCTGGAAGTGC-3′	5′-CCGGGTTATGCTGGTTGTA-3′	137
GAPDH	5′-ATCACCATCTTCCAGGAGCGA-3′	5′-CCTTCTCCATGGTGGTGAAGAC-3′	112

### ChIP-qPCR

Chromatin immunoprecipitation (ChIP) assays were performed according to manufacturer’s instructions using the ChIP assay kit from Thermo Scientific and the NF-κB antibody from Abcam. Briefly, DNA and proteins were cross-linked by the addition of formaldehyde (1% final concentration) 10 min before harvesting, and crosslinking was terminated by the addition of glycine solution for 5 min at room temperature. After that, the cells were scraped off the plates, and resuspended in PBS with lysis cocktail (1% final concentration). The DNA was then sheared into 0.5–1 kbp fragments using sonication at 20% amplitude, seven times, each for 30 s. After centrifugation, the supernatant was precleared by incubation with Protein A/G beads, adsorbed with salmon sperm DNA at 4°C. The cleared lysates were then incubated overnight with NF-κB antibody. Immune complexes were precipitated with protein A/G beads. Pre-immunized rabbit serum was used as a negative control, and the supernatant of this reaction was used as an input control. Immunoprecipitated samples were incubated at 65°C for 12 h to reverse the crosslink. DNA was extracted using a DNA extraction kit (CoWin Biotech, Beijing, China) and qPCR was performed with the following primers using the SYBR Premix Ex Taq II kit (Takara, Dalian, China): *KCNN4*-F: 5′-TCATCACTGCGAGCACTTGT-3′ *KCNN4*-R: 5′-CGAAACCCAATACGTGTAGACA-3′. A melting curve analysis was performed at the end of target gene amplification. For relative quantification, 2^-ΔΔCt^ was used to calculate the fold change in gene expression. All of the experiments were performed in triplicate.

### Plasmids and recombinants

The plasmids including pGL3-basic, pRL-TK and pGL3-promoter used for luciferase reporter gene expression analysis were purchased from Promega Ltd. A 127 bp fragment comprising -585 to -459 bp upstream of the *KCNN4* transcription start site (TSS), which contained the predicted NF-κB binding site (CCATACAGGG), was amplified and inserted into the pGL3-promoter vector to construct pGL3 - 585/-459 vector. Additionally, pGL3 - 585/-459-M vector with a mutated NF-κB binding site (CCCCGGAGGG) in the *KCNN4* regulatory region was constructed. Key regions in all constructs were verified by DNA sequencing.

### Reporter gene assays

TAMs with high endogenous expression of NF-κB were allowed to grow to 60% confluency in 24-well dishes. After 24 h, pGL3 - 585/-459, pGL3 - 585/-459-M and pGL3-basic were transfected into TAM cells using Lipofectamine™ 2000 reagent and incubated for 24 h. Cells were washed twice, suspended in 100 μl reporter lysis buffer (Promega) and luciferase activity measured using the dual luciferase reporter assay system and a GloMax 20/20 luminometer (Promega, Madison, Wisconsin, USA) according to the manufacturer’s protocol. The Renilla luciferase vector pRL-TK (Promega, Madison, Wisconsin, USA) was co-transfected to standardize transfection efficiency in each experiment.

### Immunofluorescence staining

For immunofluorescence staining, the specimens were incubated with mouse anti-hCD68 mAb (diluted 1:100), rabbit anti-hIL-6 Ab (diluted 1:100) and rabbit anti-hIL-8Ab (diluted 1:100) at 4°C overnight. Secondary staining with Alexa-Fluor-555 conjugated donkey anti-rabbit and Alexa-Fluor-488 conjugated goat anti-mouse secondary antibodies was carried out at room temperature for 60 min, followed by DAPI nuclear counterstaining for 10 min. Images were taken with a Zeiss LSM 700 laser scanning microscope (Carl Zeiss) with a core data acquisition system (Applied Precision). For control experiments, primary antibody was substituted with normal rabbit serum.

### Statistics

Statistical analyses were performed using SPSS 13.0 (SPSS Inc, USA). All data are present as the mean ± S.D. Unpaired Student’s t test and one-way ANOVA were used, as appropriate, to assess the statistical significant of differences between two groups and three or more groups respectively. χ^2^ test was applied to analyze the relationship between IL-6 and IL-8 double-positive TAMs counts and clinicopathologic features. Kaplan–Meier survival curves were plotted and log-rank test was carried out. In all cases, a value of p < 0.05 was accepted as significant.

## Results

### THP-1 cells differentiate into M2 macrophages with PMA treatment

**Figure 1:**
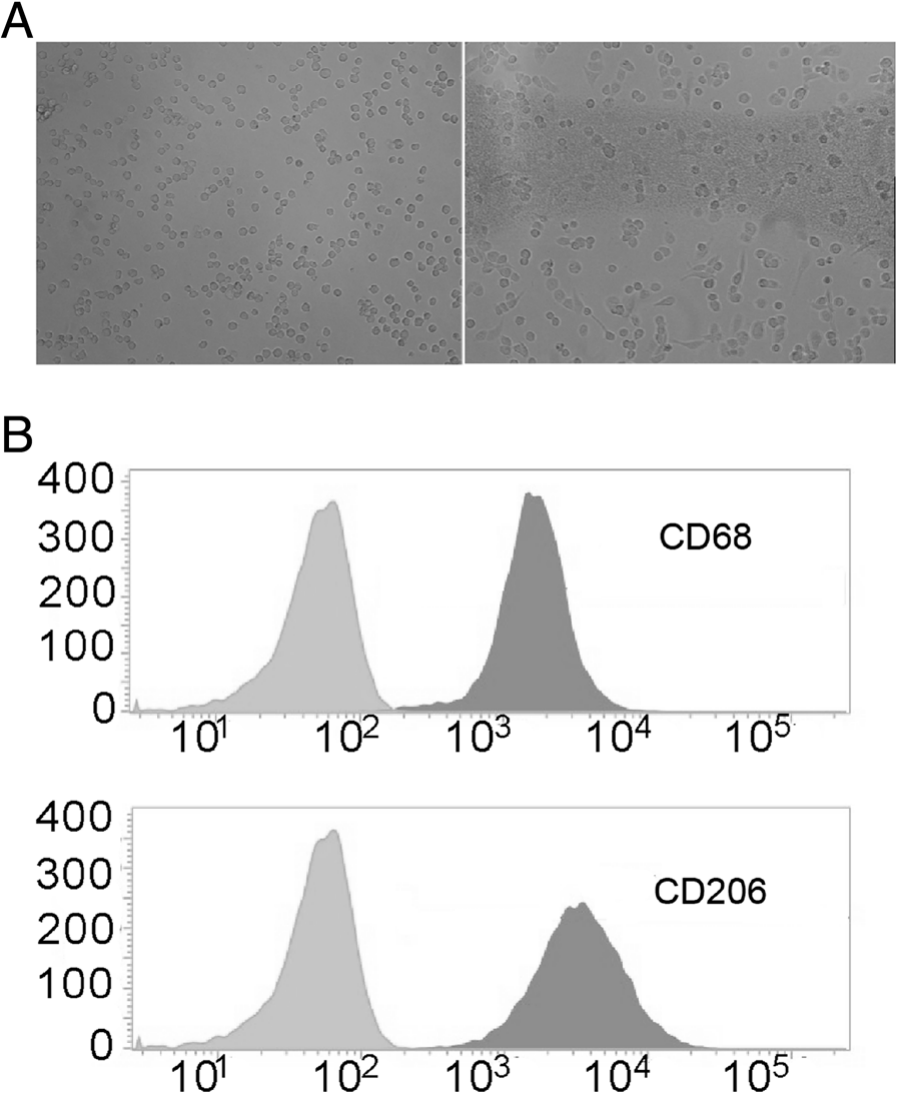
**THP-1 cells differentiate to M2 macrophages with PMA treatment. A)** Normal conditions of THP-1 (left), and treated with PMA 320 nM for 6 h with addition of IL-4 20 ng/ml for 18 h (right). **B)** PMA/IL-4 treated THP-1 cells showed significant induction of CD68 (a marker of macrophage differentiation) and CD206 (a marker of TAMs/M2 macrophages).

M2 macrophages are a type of TAM and are activated by interleukin-4 (IL-4) produced by CD4^+^ T cells. THP-1 cells are a human monocyte cell line often used for macrophage differentiation. THP-1 cells were grown in suspension, then treated with PMA (320 nM/1 × 10^6^ cells) for 6 h with subsequent addition of IL-4 (20 ng/ml) for 18 h (total of 24 h). The cells became larger and adherent, and exhibited pseudopodia. (Figure 1A). Moreover, PMA-treated THP-1 cells expressed CD68 and CD206, two surface markers of TAMs (M2 macrophages) (Figure 1B).

### PRL-3 induces the expression of KCNN4 in TAMs via TNF-α

**Figure 2:**
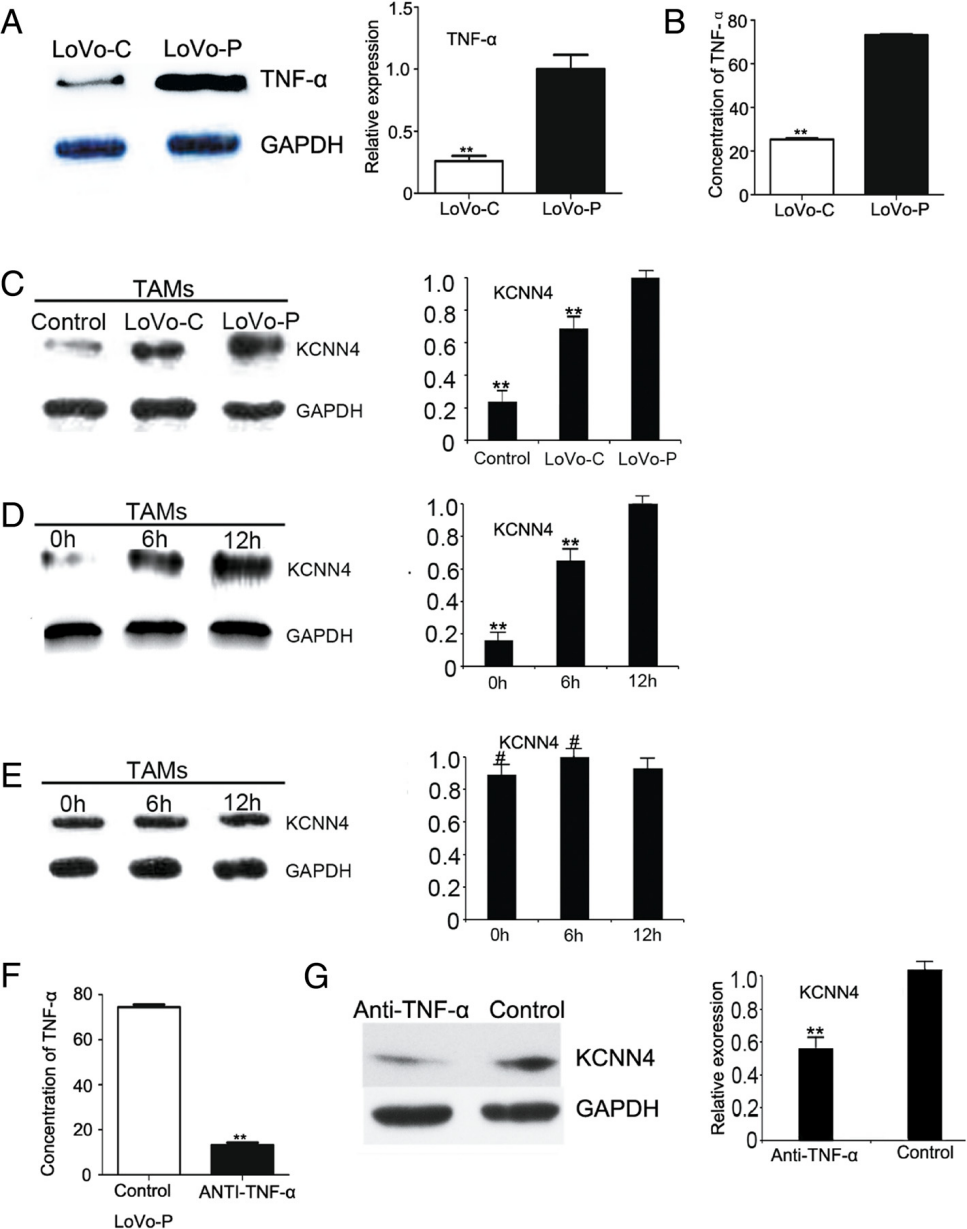
**PRL-3 induces the expression of KCNN4 by TAMs via TNF-α. A)**The expression of TNF-α in LoVo-P cells and LoVo-C cells was detected by western blot. **B)** TNF-α expression in the culture medium of LoVo-P and LoVo-C cells was detected by ELISA. **C)** Western blotting for KCNN4 in TAMs (Control) and TAMs cocultured with LoVo-C and LoVo-P cells. Bars correspond to the mean ± SD, **p < 0.01, compared with LoVo-P cells. **D)** TAMs were cocultured with LoVo-P cells for 0, 6, 12 h to detect the expression of KCNN4. Bars correspond to the mean ± SD, **p < 0.01, compared with TAMs cocultured for 12 h. **E)** TAMs were cocultured with LoVo-C cells for 0, 6, 12 h to detect the expression of KCNN4. Bars correspond to the mean ± SD, #p > 0.01, compared with TAMs cocultured for 12 h. **F)** Anti-TNF-α was added into the coculture system, and ELISA was used to detect the levels of TNF-α in the medium. Bars correspond to the mean ± SD, **p < 0.01, compared with LoVo-P cells without anti-TNF-α (Control LoVo-P). **G)** Western blot was used to detect the expression of KCNN4 of TAMs with anti-TNF-α in the coculture medium. Bars correspond to the mean ± SD, **p < 0.01, compared with Control.

Our previous research has demonstrated that PRL-3 can induce LoVo cells to secrete TNF-α and enhance the expression of KCNN4 through activation of the NF-κB pathway in an autocrine manner. Western blot was used to detect TNF-α expression in LoVo-P and LoVo-C cells (Figure 2A). ELISA was used to detect TNF-α in the culture medium (Figure 2B). The results showed that TNF-α was highly expressed in LoVo-P cells and was present in the culture medium. To determine whether PRL-3 could induce KCNN4 expression in TAMs through TNF-α in a paracrine manner, LoVo-P and LoVo-C cells were cocultured with TAMs in transwell chambers. The expression of KCNN4 in TAMs was significantly increased after coculture with LoVo-P cells (Figure 2C). Moreover, compared with LoVo-C cells, coculture of TAMs with LoVo-P cells for 6 h and 12 h caused a time-dependent increase in TAM KCNN4 (Figure 2D and E). To determine whether this increase in expression was dependent on the paracrine effects of TNF-α production by LoVo-P cells, TNF-α was neutralized by addition of anti-TNF-α to the coculture system (Figure 2F). This resulted in reduced expression of KCNN4 in TAMs (Figure 2G).

### NF-κB is capable of binding to the KCNN4 gene promoter

**Figure 3:**
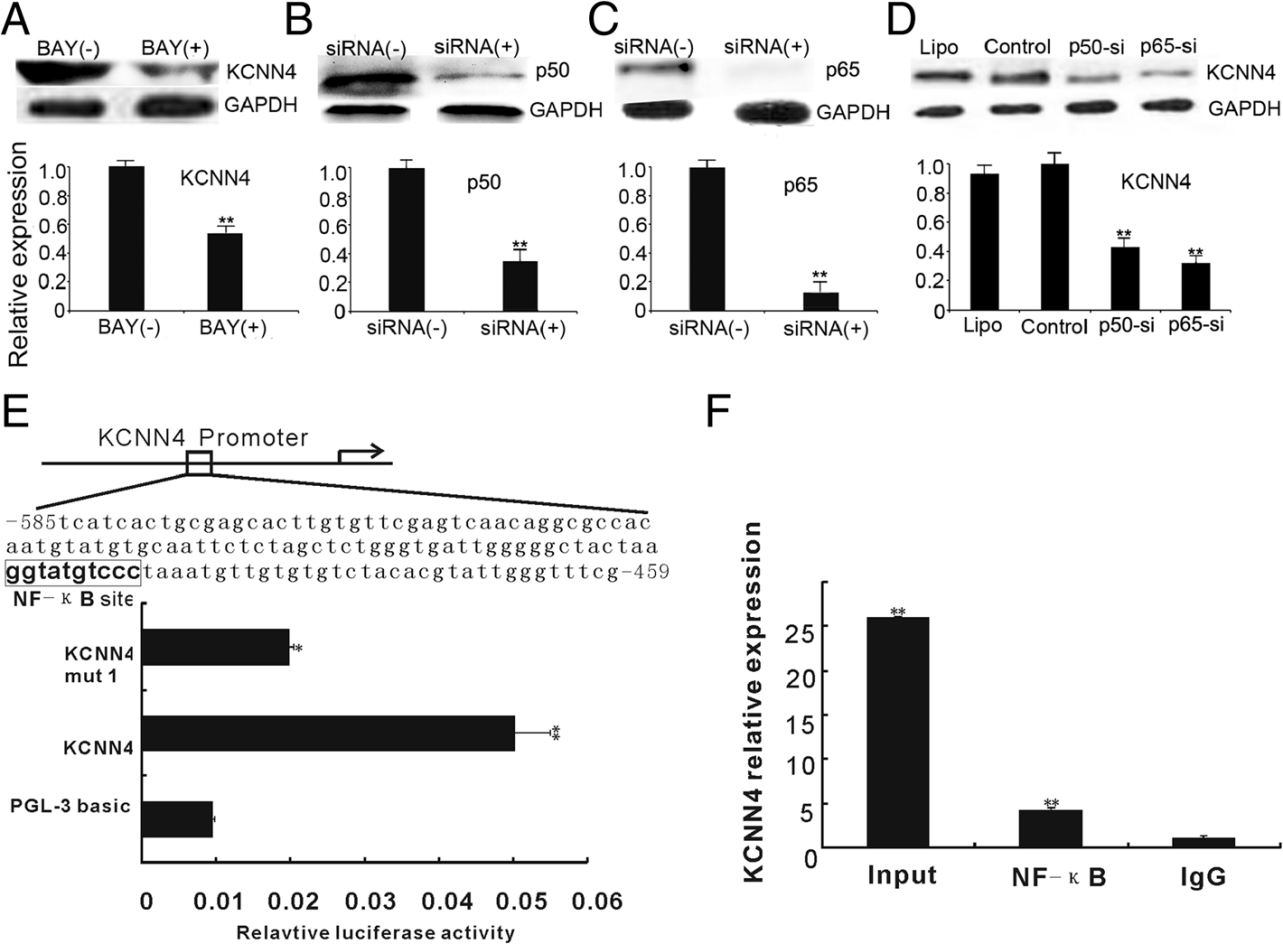
**NF-κB is capable of binding to the KCNN4 gene promoter. A)** Western blotting for KCNN4 of TAMs that were pretreated with or without BAY11-7082. Bars correspond to the mean ± SD, **p < 0.01, compared with no BAY11-7082 treatment. **B** and **C)** siRNAs were used to specifically inhibit p50 and p65. Bars correspond to the mean ± SD, **p < 0.01, compared with Control (non-transfected). **D)** Western blotting for KCNN4 of TAMs cocultured with LoVo-P cells (Control), and TAMs pretreated with Lipo2000(Lipo), p50-siRNA (p50-si), p65-siRNA (p65-si) before coculturing with LoVo-P cells. Bars correspond to the mean ± SD, **p < 0.01, compared with Control (non-transfected) and Lipofectamine 2000. **E)** Luciferase reporter assay demonstrated the influence of NF-κB on *KCNN4* promoter activity. TAMs were cotransfected with *KCNN4*-promoter-luciferase plus pRL-TK-luciferase; *KCNN4*-promoter (mut) plus pRL-TK-luciferase; or pGL-3-basic plus pRL-TK-luciferase. Luciferase activity in cell extracts was analyzed by the Dual-Luciferase Reporter Assay System and normalized using pRL-TK-luciferase activity in each sample. Bars correspond to the mean ± SD, *p < 0.05, **p < 0.01, compared with TAMs transfected with pGL-3-basic. **F)** ChIP-qPCR assay confirmed that the transcription factor NF-κB can specifically bind to the regulatory region of *KCNN4* in TAMs. Bars correspond to the mean ± SD, **p < 0.01, compared with isotype-matched IgG control (IgG).

To examine whether the expression of KCNN4 in TAMs was induced by PRL-3 through the NF-κB signaling pathway, we pretreated TAMs with BAY11-7082, which is known to inhibit the activity of NF-κB, and we cocultured the TAMs with LoVo-P cells. The expression of KCNN4 was clearly decreased when NF-κB activity was suppressed (Figure 3A). Furthermore, specific siRNAs were used to silence the expression of p50 and p65 (Figure 3B and C), as they are important for NF-κB binding. After coculture of siRNA-treated TAMs with LoVo-P cells for 12 h, the expression of KCNN4 was reduced by 62% and 53% after p50 and p65 were silenced, respectively (Figure 3D). To further determine whether NF-κB regulates KCNN4 expression through binding to its promoter, we evaluated transcription factor binding sites in *KCNN4* regulatory regions using the JASPAR database ( http://jaspar.genereg.net/cgi-bin/jaspar_db.pl). An NF-κB recognition site (CCATACAGGG) was discovered in the 5′ regulatory region of the KCNN4 gene, suggesting that expression of KCNN4 may be regulated by the transcription factor NF-κB. To further explore whether NF-κB regulates *KCNN4* expression through binding to its promoter, we performed transient transfection assays in TAMs with *KCNN4*/pGL3 - 585/-459 reporters. The results showed that the luciferase activity of the reporter system in transfected cells was markedly higher than that in parental TAMs and TAMs transfected with the *KCNN4*/pGL3 - 585/-459-M mutant, in which the NF-κB binding site was mutated via PCR-directed mutagenesis (Figure 3E). Moreover, ChIP assays were used to determine whether RelA/p65, which is one of the subunits of NF-κB, binds to the promoter of *KCNN4*. ChIP was performed using an anti-RelA/p65 antibody. A 127 bp fragment of the *KCNN4* sequence was amplified, indicating that the RelA/p65 transcription factor can directly bind to the specific promoter region of the *KCNN4* gene (Figure 3F). Together, these results indicate that NF-κB directly binds to the promoter of *KCNN4* and regulates promoter activity.

### TAMs promote the invasive activity of LoVo-P cells through KCNN4

**Figure 4:**
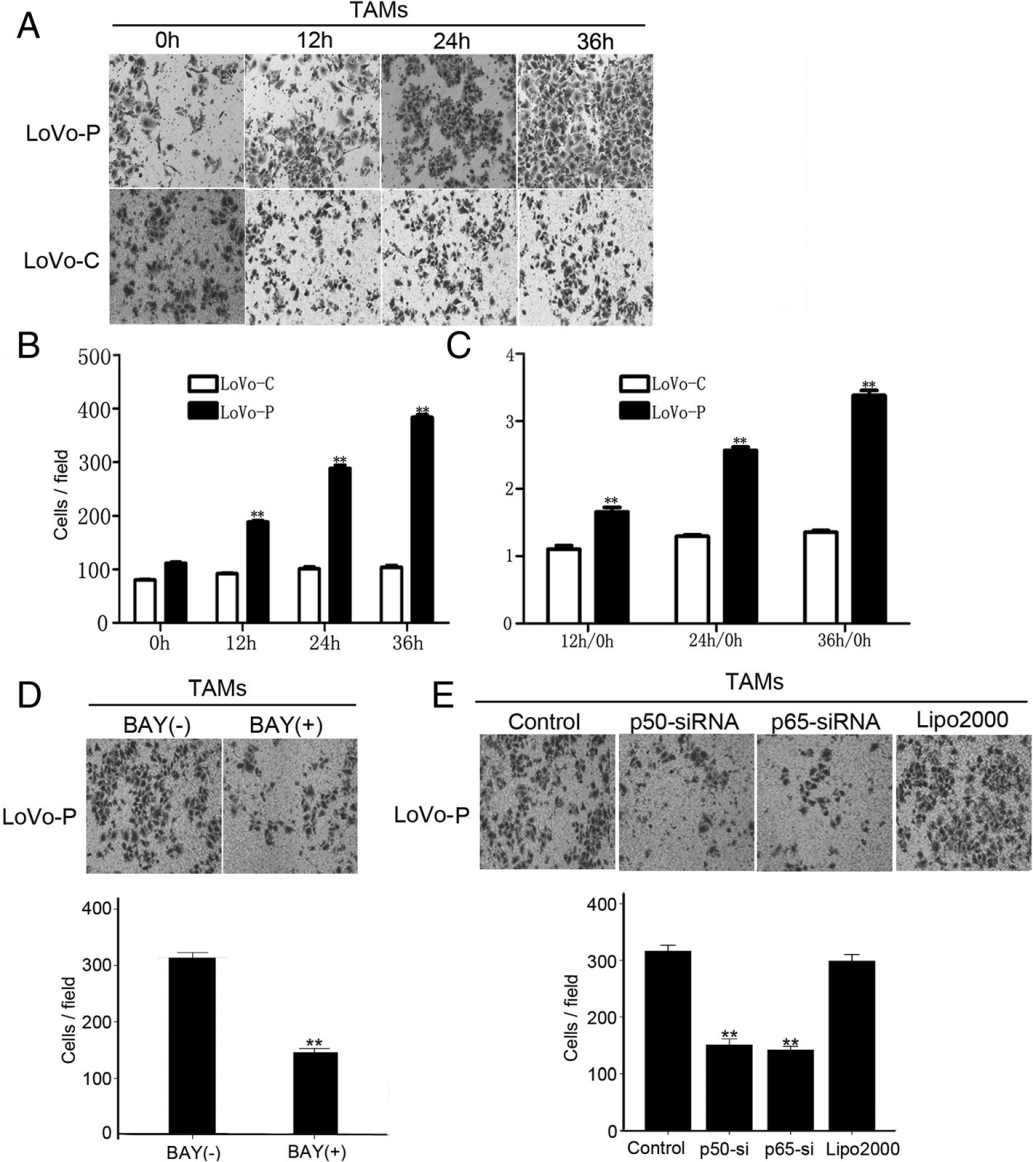
**TAMs promote the invasive activity of LoVo-P cells through KCNN4. A)** Transwell chamber assays for LoVo-P cells and LoVo-C cells cocultured with TAMs for 0, 12, 24, or 36 h. **B)** Bars correspond to the mean ± SD, **p < 0.01. The numbers of cells passed through the Matrigel matrix. **C)** The ratio of LoVo cells cocultured with/without TAMs. **D)** TAMs were pretreated with or without BAY11-7082, and then cocultured in transwell chamber assays with LoVo-P cells. Bars correspond to the mean ± SD, **p < 0.01, compared with no BAY11-7082 treatment. **E)** Transwell chamber assays for LoVo-P cells cocultured with TAMs (Control) or cocultured with TAMs pretreated with p50-siRNA, p65-siRNA and Lipofectamine 2000. Bars correspond to the mean ± SD, **p < 0.01, compared with Control (non-transfected) and Lipofectamine 2000.

To further investigate the role of TAMs in the invasive behavior of LoVo cells, we cocultured TAMs with LoVo-P cells or with LoVo-C cells. LoVo cell invasion was detected using a transwell chamber and Matrigel. When interacting with TAMs, LoVo-P cells showed greater invasive activity than LoVo-C cells (Figure 4A and B). To exclude any differences in the invasive activity that may be caused by the cells themselves, we compared the invasiveness of LoVo-P cells cocultured with TAMs for 12 h, 24 h or 36 h with LoVo-P cells that were not cocultured with TAMs, and found that the invasiveness of cocultured LoVo-P cells was enhanced by 1.7-fold, 2.4-fold, and 3.3-fold, respectively. However, the invasiveness of cocultured LoVo-C cells did not show a significant increase compared with LoVo-C cells that were not cocultured with TAMs (Figure 4C). Moreover, BAY11-7082 was used to inhibit the NF-κB signaling pathway in TAMs, which were then cocultured with LoVo-P. Interestingly, the invasiveness of the LoVo cells decreased significantly as the level of KCNN4 in TAMs was reduced (Figure 4D). Moreover, when p50-siRNA and p65-siRNA were used to inhibit the expression of KCNN4 channels in TAMs, we found that the invasive activity of cocultured LoVo-P cells was significantly inhibited (Figure 4E). These results demonstrate that the invasive ability of LoVo cells induced by PRL-3 is regulated by the expression of KCNN4 channels in TAMs.

### TAMs promote the invasive activity of LoVo-P cells via IL-6 and IL-8

**Figure 5:**
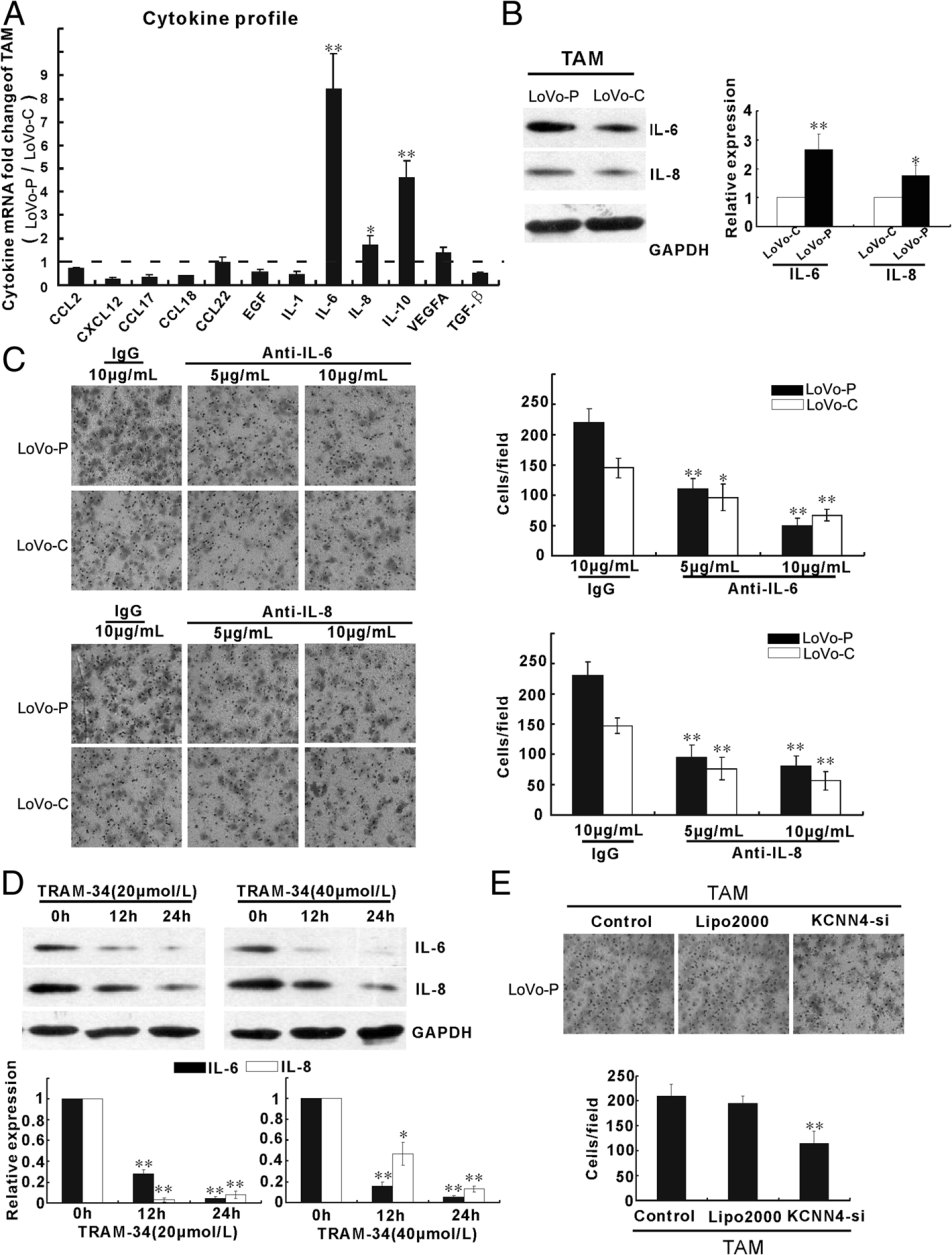
**M2-polarized TAMs enhance the invasive activity of LoVo cells induced by PRL-3 via IL-6 and IL-8. A-B)** The expression of cytokine profile of TAMs cocultured with LoVo-P cells and LoVo-C cells determined by qRT-PCR **(A)**, and protein by western blotting **(B)**. **C)** Cell invasion of LoVo-P/LoVo-C cells was evaluated after plating the cells on the upper cell culture inserts, with culture medium TAMs plated in the lower chambers in the presence of anti-IL-6/IL-8 antibody at 5 or 10 μg/ml, or an isotype-matched IgG control (IgG). **D)** The expression ratios for IL-6 and IL-8 in conditioned medium of TAMs wa determined by western blotting. GAPDH was used as a loading control. **E)** Similar to **(C)**, LoVo-P cells were cocultured with TAMs that were untreated (Control), mock transfected (Lipofectamine 2000), or transfected with *KCNN4*-si. Bars correspond to the mean ± SD, *p < 0.05, **p < 0.01, compared with Control.

To further explore the mechanism by which TAMs promote the invasion of LoVo cells, qRT-PCR was performed to screen a panel of cytokines related to TAMs. Once the TAMs were cocultured with LoVo-P cells, the expression levels of *IL-6* and *IL-8* mRNA were higher than those in TAMs cocultured with the LoVo-C cells (Figure 5A). Additionally, western blotting showed that IL-6 and IL-8 protein levels were significantly increased after TAMs were cocultured with LoVo-P cells (Figure 5B). To further validate the important role of IL-6 and IL-8 in LoVo cancer cell invasion induced by PRL-3, anti-IL-6 antibody and anti-IL-8 antibody were used to neutralize IL-6 and IL-8 function. The addition of the IL-6 and IL-8 antibodies to the coculture system of TAMs and LoVo-P cells reduced the number of invasive cancer cells in a dose-dependent manner; an isotype-matched IgG at 10 μg/ml did not have similar effects (Figure 5C). To further explore whether KCNN4 channels contribute to the upregulation of IL-6 and IL-8, *KCNN4*-siRNAs were transfected into the TAMs. This resulted in the reduction of IL-6 and IL-8 (Figure 5D) and a concurrent reduction in the number of invasive cancer cells (Figure 5E). These results suggest that both IL-6 and IL-8 secreted by TAMs promoted the invasiveness of LoVo cells induced by PRL-3 through the activation of KCNN4 channels.

### TAMs express IL-6 and IL-8 in colorectal cancer

**Figure 6:**
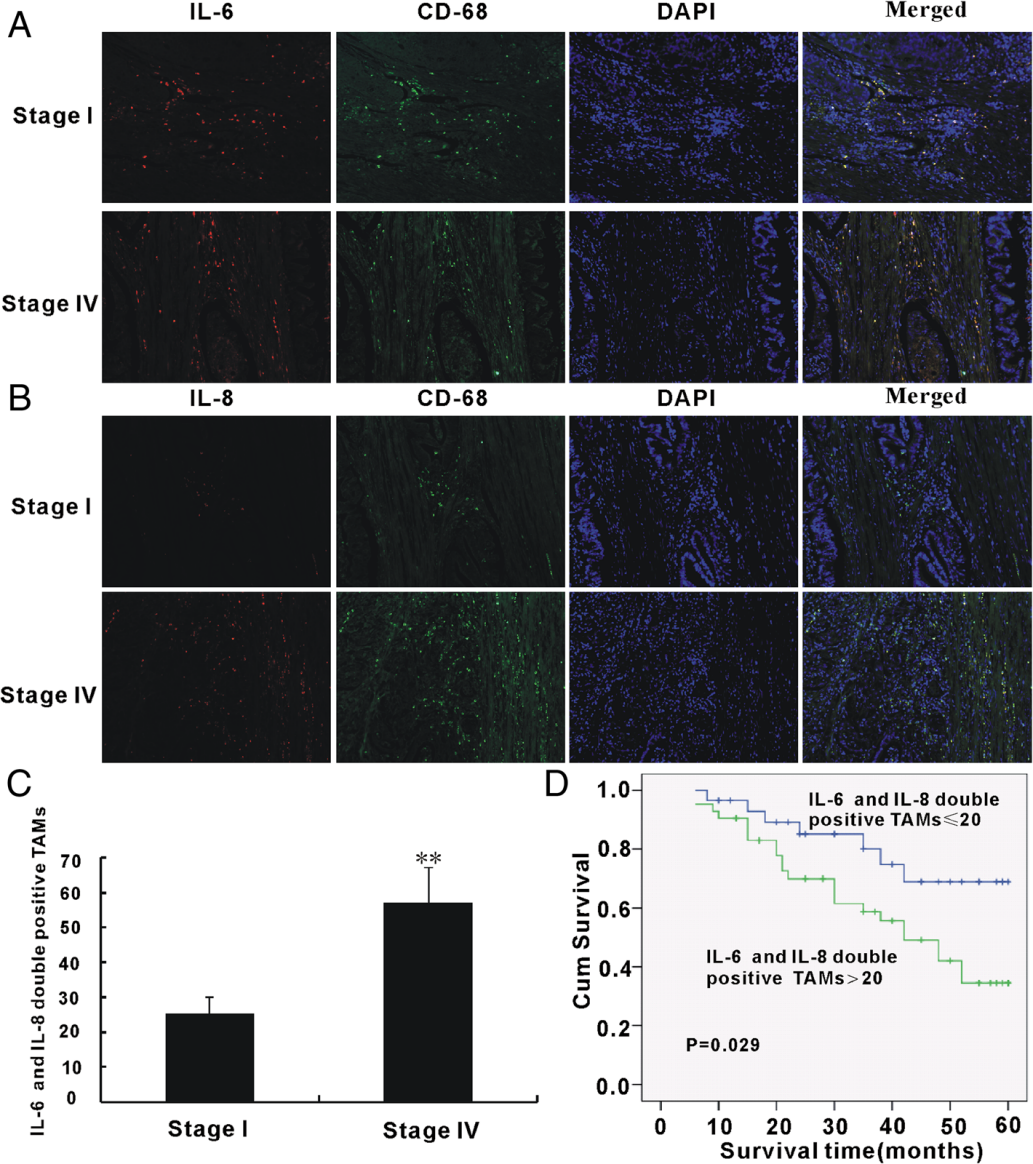
**Immunostaining for TAMs, IL-6 and IL-8 in CRC tissues from early to late stage. A** and **B)** Confocal microscopy for immunostaining of stages I and IV CRC tissues with CD68 antibody (green), anti-IL-6 (A, red) and anti-IL-8 (B, red). Cell nuclei were counterstained with DAPI (original magnification, ×200), (images of high resolution are in the Additional files 1 and 2). **C)** Quantitative analysis of number of TAMs per field. **p < 0.01, compared with stage IV. **D)** Kaplan–Meier survival curve of patients with colorectal cancer with lower (≤20 per view of field, n = 29) and higher IL-6 and IL-8 double positive TAMs counts (>20 per view of field, n = 42; p < 0.05).

To further explore the expression of IL-6 and IL-8 in colorectal carcinogenesis, immunofluorescence staining was used to detect the distribution of IL-6 and IL-8 in CRC samples. Interestingly, we observed that IL-6 and IL-8 were only expressed in the stroma and not in the tumor cells. Next, we further tested whether IL-6 and IL-8 positive cells in the stroma were TAMs. By performing immunofluorescence staining of IL-6, IL-8 and CD68, we demonstrated that many IL-6 and IL-8 double-positive cells in the stroma were also CD68 positive (Figure 6A and B, Additional files 1 and 2). These results suggest that IL-6 and IL-8 are produced in the stroma of colorectal cancer cells and that TAMs are the major source of stromal IL-6 and IL-8. Furthermore, we also compared the number of IL-6 and IL-8 double positive TAMs in metastatic CRC (stages III and IV) and early-stage CRC (stages I and II). The number of IL-6 and IL-8-expressing TAMs in metastatic CRC was 2.28-fold higher than those in early-stage CRC (Figure 6C). To further evaluate the clinical relevance of TAMs in CRC, we analyzed their association with the clinicopathologic status of patients (Table 2). No significant correlation was observed between the number of TAMs and age or tumor site of the patients. However, the number of TAMs was closely associated with clinical staging and lymph node metastasis of the patients. Patients with tumors at advanced clinical stages (stages III and IV; p < 0.001) and lymph node metastasis (p < 0.001) expressed higher levels of IL-6 and IL-8 double-positive TAMs, suggesting that IL-6 and IL-8 double-positive TAMs are related to cancer progression. A Kaplan–Meier survival curve with a median follow-up period of 50 months demonstrated that patients with a low IL-6 and IL-8 double-positive TAM count (≤20) survive significantly longer than those with high IL-6 and IL-8 double-positive TAM counts (>20) (Figure 6D; p = 0.029).

**Table 2:** Correlation of IL-6 and IL-8 double positive TAMs counts with clinicopathological status in 71 cases of colorectal cancer patients

**Double positive TAMs counts**	**≤20**	**>20**	**P value**
Age	67.5±5.9	68.5±6.9	
Gender			
Male	15	25	0.515
Female	14	17	
Clinical stage			
I- II	22	12	<0.001
III-IV	7	30	
Lymph node metastasis			
Negative	20	8	<0.001
Positive	9	34	
Site			
Colon	15	24	0.652
Rectum	14	18	

## Discussion

The tumor microenvironment has been shown to be composed of both of tumor cells and mesenchymal cells, and that the microenvironment itself is involved in tumorigenesis [12]. Tumor-associated macrophages, or TAMs, are macrophages that are located in the tumor environment. There are two types of macrophages, M1 and M2. M1 macrophages have antitumor activities and can produce TNF-α, whereas M2 macrophages are a type of TAM that can produce TGF-β and express CD68 and CD206 surface markers. We used PMA to induce the transformation of THP-1 monocytes into M2 macrophages according to described methods [13]. TAMs contribute to tumor progression by releasing a variety of cytokines, such as VEGF, PDGF and IL-10 [14]. In the tumor microenvironment, autocrine and paracrine loops, controlled by cytokines and receptors, have been observed between tumor-associated macrophages and tumor cells during tumor initiation, promotion, and metastasis [15,16]. Our previous studies have demonstrated that PRL-3 can promote the proliferation and metastasis of CRC cells through the autocrine secretion of TNF-α, which induces KCNN4 channel expression by activating the NF-κB signaling pathway. Considering that TNF-α could act as an autocrine and paracrine cytokine to promote the proliferation and metastasis of tumor cells [17], we speculated that there might be a paracrine loop between CRC cell and TAMs in the tumor microenvironment that is controlled via TNF-α. In this study, we showed that TAMs participate in the progression of CRC induced by PRL-3 through the TNF-α mediated-secretion of IL-6 and IL-8 in a paracrine manner. Moreover, such regulation could be inhibited by TRAM-34, a KCNN4 channel-specific inhibitor.

To our knowledge, this is the first report indicating that KCNN4 channels participate in PRL-3 induced secretion of IL-6 and IL-8 by TAMs. Previous studies have demonstrated that KCNN4 channels belong to the Ca^2+^-activated potassium channel superfamily, and the activation of these channels is dependent on conformational changes in calcium calmodulin [18]. KCNN4 channels are mainly expressed in peripheral tissues, including the hematopoietic system, colon, lung and pancreatic tissue, and play an important role in the transport of substances [19]. Previous research has also revealed that KCNN4 channels regulate cell cycle progression and cell growth in human endometrial cancer and prostate cancer cells [20,21]. Our data showed that when LoVo-P cells were cocultured with TAMs, the expression of KCNN4 channels was significantly increased, indicating that transcriptional mechanisms are likely to be responsible for the increased KCNN4 expression. A previous study revealed that activation protein-1 (AP-1) could regulate KCNN4 channel expression in T-cell activation [22]. Our research demonstrated that LoVo-P cells could release TNF-α and subsequently regulate the KCNN4 expression of TAMs in a paracrine manner. Consistent with our hypothesis, ChIP-qPCR and reporter gene assays indicated that NF-κB was required for transcription of the *KCNN4* gene and mediated the transcriptional activation of KCNN4 channel expression in TAMs when they were cocultured with LoVo-P cells.

Although many studies have highlighted the role of TAMs in tumor metastasis, we still questioned whether TAMs could enhance the metastasis of tumor cells induced by PRL-3. In this study, when LoVo-P cells were cocultured with TAMs, invasion was significantly enhanced. Moreover, we demonstrated that PRL-3 is important for the ability of TAMs to enhance LoVo cell invasiveness, as LoVo-P cells cocultured with TAMs exhibited increased invasiveness compared with LoVo-P cells that were not cocultured with TAMs. LoVo-C cells did not exhibit the same features. Additionally, when TAMs were pretreated with p50/p65-siRNA or BAY11-7082, the invasive ability of LoVo-P cells was inhibited and the expression of KCNN4 channels was decreased. It is possible that the KCNN4 channels of TAMs enhanced the PRL-3-induced metastasis of CRC cells.

Previous studies have shown that potassium channels can regulate the cytokine secretion of human activated macrophages [23]. We therefore tested whether KCNN4 channels could regulate the cytokine secretion of TAMs. Our data showed that once LoVo-P cells were cocultured with TAMs, the secretion of IL-6 and IL-8 was significantly increased. Moreover, it is notable that the secretion of IL-10 was also increased significantly. IL-10 is produced mainly by T cells and macrophages and has a role in immunoregulation. It has been shown that IL-10 can inhibit the expression of MHC molecules and costimulatory molecules, and can reduce antigen-presentation [24]. Besides, IL-10 can also impair secondary CD8^+^ T cell responses and thus inhibit tumor immunity [25]. In addition, it has been shown that TAMs in the tumor microenvironment not only secret inflammatory mediators but also have immunoregulatory effects [26]. We are therefore curious as to whether IL-10 produced by TAMs may contribute to the immunosuppressive tumor environment, and this feature of TAMs will be studied in our future research. Next, we explored whether KCNN4 channels could regulate IL-6 and IL-8 expression. When TAMs were pretreated with TRAM-34, the expression of IL-6 and IL-8 was significantly decreased, compared with controls, when the TAMs were cocultured with LoVo-P cells. These observations suggest that KCNN4 channels have the ability to regulate TAM secretion of IL-6 and IL-8.

The mechanism by which KCNN4 channels regulate IL-6 and IL-8 expression of TAMs remains to be investigated. It is known that KCNN4 channels in lymphocytes can maintain a hyperpolarized membrane potential, thereby facilitating and maintaining the intracellular Ca^2+^ levels required for cell proliferation and gene expression [27,28]. Recent studies have revealed that intracellular Ca^2+^ levels regulate the secretion of TNF-α and IL-6, which play important roles in inflammation [29]. However, another previous study revealed that a calcium ionophore can inhibit IL-6 and IL-8 expression in Jurkat T-cells [30]. Further studies are needed to verify whether the intracellular Ca^2+^ levels of TAMs can regulate the expression of IL-6 and IL-8 and to determine the underlying mechanism.

Cancer-associated inflammation is known to affect the proliferation, angiogenesis, and metastasis of tumor cells [31]. For example, the activation of NF-κB triggers the production of the inflammatory chemokine IL-8 by tumor cells [32]. Inhibition of the IL-6 signaling pathway inhibits tumor development in a colitis-associated carcinogenesis model [33]. In our research, the levels of TAM-derived IL-6 and IL-8 were significantly increased upon coculture with LoVo-P cells. Using the transwell-invasion system, IL-6 and IL-8 significantly enhanced the ability of LoVo-P cells to metastasize, and this response was attenuated by IL-6 and IL-8 antibodies. These results suggest that TAM-derived IL-6 and IL-8 induced by PRL-3 affect the metastasis of tumor cells in a paracrine manner.

Further confirming this result, and extending it to human disease, we demonstrated that IL-6 and IL-8 were mainly expressed by TAMs in CRC tissues. Studies have shown that serum levels of IL-6 and IL-8 were higher in CRC patients versus controls, indicating that IL-6 and IL-8 may be potential targets for CRC [34]. Furthermore, our data also demonstrated that a high density of TAMs expressing IL-6 and IL-8 was positively correlated with tumor stage. Therefore, targeting therapies against IL-6, IL-8, and KCNN4 channels of TAMs may prevent CRC liver metastasis through a number of mechanisms, including both metastasis prevention and anti-inflammatory effects.

## Conclusions

Our study demonstrated that IL-6 and IL-8 activity in TAMs promoted the tumorigenesis of CRC and served as a mediator of epithelial-stromal interaction. We also revealed the presence of paracrine loops between PRL-3 and TAMs, which function via TNF-α in the tumor microenvironment. This regulatory pathway may be a potential target for the development of new therapeutic strategies for patients with CRC liver metastasis.

## Abbreviations

PRL-3: Phosphatase of regenerating liver-3; KCNN4: Intermediate-conductance Ca^2+^-activated K^+^ channel; CRC: Colorectal cancer; TAM: Tumor associated macrophage; MMP: Matrix metalloproteinase; EGFR: Epidermal growth factor receptor; PMA: Phorbol-12-myristate-13-acetate; TRAM-34: 1-[(2-chlorophenyl) diphenylmethyl]-1H-pyrazole; TNF-α: Tumor necrosis factor-alpha.

## Competing interests

The authors declare that they have no competing interests.

## Authors’ contributions

ZHC designed the research; HYX, WL carried out the vector construction and stable cell line generation; HYX, WL, YZ, carried out the flow cytometry, cell coculture, western blotting, RT-PCR, ELISA, ChIP-qPCR, reporter gene assays; LL collected the patients’ data and carried out immunofluorescence staining. XXL carried out the invasion assay; HW and QSL carried out the statistical analysis; HYX and WL wrote the paper. All of the authors have been involved in revising the manuscript and have given final approval of the version to be published.

## Pre-publication history

The pre-publication history for this paper can be accessed here:

http://www.biomedcentral.com/1471-2407/14/330/prepub

## Supplementary Material

Additional file 1 The original high solution images of immunostaining for TAMs and IL-6 in CRC tissues from early to late stage. Click here for file

Additional file 2 The original high solution images of immunostaining for TAMs and IL-8 in CRC tissues from early to late stage. Click here for file

## References

[B1] Schafer M Werner S Cancer as an overhealing wound: an old hypothesis revisited Nat Rev Mol Cell Biol 2008 9 8 628 638 10.1038/nrm2455 18628784

[B2] Mantovani A Molecular pathways linking inflammation and cancer Curr Mol Med 2010 10 369 373 10.2174/156652410791316968 20455855

[B3] Gordon S Alternative activation of macrophages Nat Rev Immunol 2003 3 23 25 10.1038/nri978 12511873

[B4] Cardoso AP Pinto ML Pinto AT Oliveura MI Pinto MT Goncalves R Relvas JB Figueiredo C Seruca R Mantovani A Mareel M Barbosa MA Oliveira MJ Macrophages stimulate gastric and colorectal cancer invasion through EGFR Y1086, c-Src, Erk1/2 and Akt phosphorylation and smallGTPase activity Oncogene 2013 10 1038 10.1038/onc.2013.154 23644655

[B5] Al-Aidaroos AQ Zeng Q PRL-3 phosphatase and cancer metastasis J Cell Biochem 2010 111 1087 1098 10.1002/jcb.22913 21053359

[B6] Jiang Y Liu XQ Rajput A Geng L Ongchin M Zeng Q Taylor GS Wang J Phosphatase PRL-3 is a direct regulatory target of TGFbeta in colon cancer metastasis Cancer Res 2011 71 234 244 10.1158/0008-5472.CAN-10-1487 21084277 PMC3064433

[B7] Lai W Chen S Wu H Guan Y Liu L Zeng Y Zhao H Jiang J Chu Z PRL-3 promotes the proliferation of LoVo cells via the upregulation of KCNN4 channels Oncol Rep 2011 26 909 917 21725609 10.3892/or.2011.1366

[B8] Hong DS Angelo LS Kurzrock R Interleukin-6 and its receptor in cancer: implications for translational therapeutics Cancer 2007 110 1911 1928 10.1002/cncr.22999 17849470

[B9] Schafer ZT Brugge JS IL-6 involvement in epithelial cancers J Clin Invest 2007 117 3660 3663 10.1172/JCI34237 18060028 PMC2096452

[B10] Ning Y Manegold PC Hong YK Zhang W Pohl A Lurje G Winder T Yang D LaBonte MJ Wilson PM Ladner RD Lenz HJ Interleukin-8 is associated with proliferation, migration, angiogenesis and chemosensitivity in vitro and in vivo in colon cancer cell line models Int J Cancer 2011 128 2038 2049 10.1002/ijc.25562 20648559 PMC3039715

[B11] Heidemann J Ogawa H Dwinell MB Rafiee P Maaser C Gockel HR Otterson MF Ota DM Lugering N Domschke W Binion DG Angiogenic effects of interleukin 8 (CXCL8) in human intestinal microvascular endothelial cells are mediated by CXCR2 J Biol Chem 2003 278 8508 8515 10.1074/jbc.M208231200 12496258

[B12] Grivennikov SI Greten FR Karin M Immunity, inflammation, and cancer Cell 2010 140 6 883 899 10.1016/j.cell.2010.01.025 20303878 PMC2866629

[B13] Tjiu JW Chen JS Shun CT Lin SJ Liao YH Chu CY Tsai TF Chiu HC Dai YS Inoue H Yang PC Kuo ML Jee SH Tumor-associated macrophage-induced invasion and angiogenesis of human basal cell carcinoma cells by cyclooxygenase-2 induction J Invest Dermatol 2009 129 1016 1025 10.1038/jid.2008.310 18843292

[B14] Jedinak A Dudhgaonkar S Sliva D Activated macrophages induce metastatic behavior of colon cancer cells Immunobiology 2010 215 242 249 10.1016/j.imbio.2009.03.004 19457576

[B15] Wu Y Garmire LX Fan R Inter-cellular signaling network reveals a mechanistic transition in tumor microenvironment Integr Biol 2012 4 1478 1486 10.1039/c2ib20044a PMC3502715 23080410

[B16] Tsujikawa T Yaguchi T Ohmura G Ohta S Kobayashi A Kawamura N Fujita T Nakano H Shimada T Takahashi T Nakao R Yanagisawa A Hisa Y Kawakami Y Autocrine and paracrine loops between cancer cells and macrophages promote lymph node metastasis via CCR4/CCL22 in head and neck squamous cell carcinoma Int J Cancer 2013 132 2755 2766 10.1002/ijc.27966 23180648

[B17] Wu S Boyer CM Whitaker RS Berchuck A Wiener JR Weinberg JB Bast RC Jr Tumor necrosis factor alpha as an autocrine and paracrine growth factor for ovarian cancer: monokine induction of tumor cell proliferation and tumor necrosis factor alpha expression Cancer Res 1993 53 1939 1944 8385577

[B18] Fanger CM Ghanshani S Logsdon NJ Rauer H Kalman K Zhou J Beckingham K Chandy KG Cahalan MD Aiyar J Calmodulin mediates calcium-dependent activation of the intermediate conductance KCa channel, IKCa1 J Biol Chem 1999 274 5746 5754 10.1074/jbc.274.9.5746 10026195

[B19] Dong H Smith A Hovaida M Chow JY Role of Ca2+-activated K+ channels in duodenal mucosal ion transport and bicarbonate secretion Am J Physiol Gastrointest Liver Physiol 2006 291 G1120 G1128 10.1152/ajpgi.00566.2005 16763288

[B20] Wang ZH Shen B Yao HL Jia YC Ren J Feng YJ Wang YZ Blockage of intermediate-conductance-Ca(2+) -activated K(+) channels inhibits progression of human endometrial cancer Oncogene 2007 26 5107 5114 10.1038/sj.onc.1210308 17310992

[B21] Lallet-Daher H Roudbaraki M Bavencoffe A Mariot P Gackiere F Bidaux G Urbain R Gosset P Delcourt P Fleurisse L Slomianny C Dewailly E Mauroy B Bonnal JL Skryma R Prevarskaya N Intermediate-conductance Ca2+-activated K+ channels (IKCa1) regulate human prostate cancer cell proliferation through a close control of calcium entry Oncogene 2009 28 1792 1806 10.1038/onc.2009.25 19270724

[B22] Ghanshani S Wulff H Miller MJ Rohm H Neben A Gutman GA Cahalan MD Chandy KG Up-regulation of the IKCa1 potassium channel during T-cell activation. Molecular mechanism and functional consequences J Biol Chem 2000 275 37137 37149 10.1074/jbc.M003941200 10961988

[B23] Qiu MR Campbell TJ Breit SN A potassium ion channel is involved in cytokine production by activated human macrophages Clin Exp Immunol 2002 130 67 74 10.1046/j.1365-2249.2002.01965.x 12296855 PMC1906495

[B24] Moore KW de Waal Malefyt R Coffman RL O’Garra A Interleukin-10 and the interleukin-10 receptor Annu Rev Immuno 2001 19 683 765 10.1146/annurev.immunol.19.1.683 11244051

[B25] Kang S Allen P Priming in the presence of IL-10 results in direct enhancement of CD8+ T cell primary responses and inhibition of secondary responses J Immunol 2005 174 5382 5389 10.4049/jimmunol.174.9.5382 15843536

[B26] Hanahan D Weinberg RA Hallmarks of cancer: the next generation Cell 2011 144 646 674 10.1016/j.cell.2011.02.013 21376230

[B27] Fanger CM Rauer H Neben AL Miller MJ Wulff H Rosa JC Ganellin CR Chandy KG Cahalan MD Calcium-activated potassium channels sustain calcium signaling in T lymphocytes. Selective blockers and manipulated channel expression levels J Biol Chem 2001 276 12249 12256 10.1074/jbc.M011342200 11278890

[B28] Spitzner M Ousingsawat J Scheidt K Kunzelmann K Schreiber R Voltage-gated K+ channels support proliferation of colonic carcinoma cells FASEB J 2007 21 35 44 17135369 10.1096/fj.06-6200com

[B29] Jeong HJ Hong SH Lee DJ Park JH Kim KS Kim HM Role of Ca(2+) on TNF-alpha and IL-6 secretion from RBL-2H3 mast cells Cellular Signal 2002 14 633 639 10.1016/S0898-6568(02)00005-0 11955956

[B30] Khalaf H Jass J Olsson PE The role of calcium, NF-kappaB and NFAT in the regulation of CXCL8 and IL-6 expression in Jurkat T-cells Int J Biochem Mol Biol 2013 4 150 156 24049670 PMC3776147

[B31] Erreni M Mantovani A Allavena P Tumor-associated macrophages (TAM) and inflammation in colorectal cancer Cancer Microenviron 2011 4 141 154 10.1007/s12307-010-0052-5 21909876 PMC3170420

[B32] Wang S Liu Z Wang L Zhang X NF-kappaB signaling pathway, inflammation and colorectal cancer Cell Mol Immunol 2009 6 327 334 10.1038/cmi.2009.43 19887045 PMC4003215

[B33] Atreya R Mudter J Finotto S Mullberg J Jostock T Wirtz S Schütz M Bartsch B Holtmann M Becker C Strand D Czaja J Schlaak JF Lehr HA Autschbach F Schürmann G Nishimoto N Yoshizaki K Ito H Kishimoto T Galle PR Rose-John S Neurath MF Blockade of interleukin 6 trans signaling suppresses T-cell resistance against apoptosis in chronic intestinal inflammation: evidence in crohn disease and experimental colitis in vivo Nat Med 2000 6 583 588 10.1038/75068 10802717

[B34] Kantola T Klintrup K Vayrynen JP Vornanen J Bloigu R Karhu T Herzig KH Näpänkangas J Mäkelä J Karttunen TJ Tuomisto A Mäkinen MJ Stage-dependent alterations of the serum cytokine pattern in colorectal carcinoma Br J Cancer 2012 107 1729 1736 10.1038/bjc.2012.456 23059742 PMC3493870

