# Supplementary figures and images for: Tumor-associated macrophage-derived IL-6 and IL-8 enhance invasive activity of LoVo cells induced by PRL-3 in a KCNN4 channel-dependent manner
Source: BMC Cancer. 2014 May 10;14:330. doi: 10.1186/1471-2407-14-330 (PMC4024187; doi:10.1186/1471-2407-14-330)

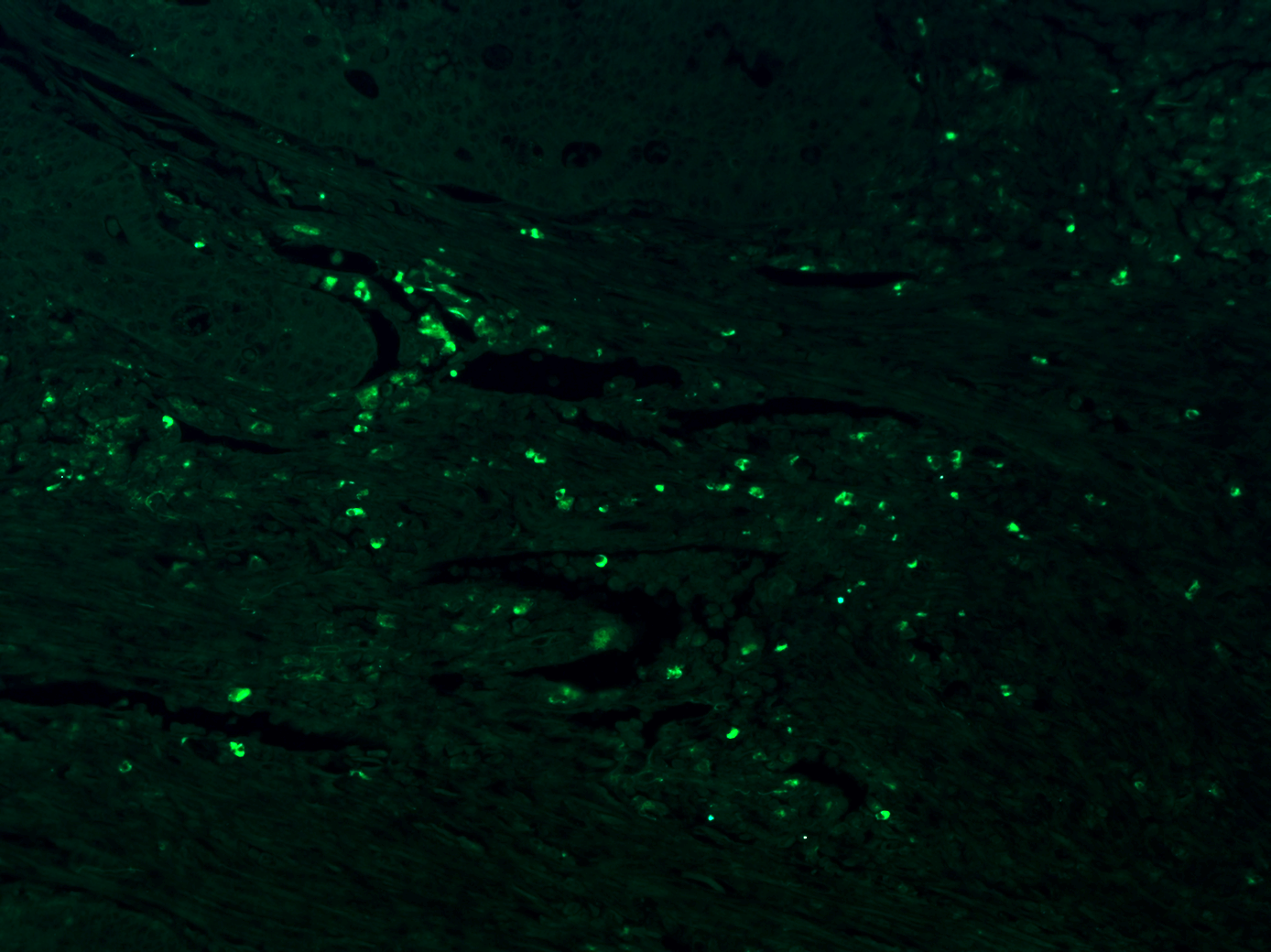

Supplement: Additional file 1 — The original high solution images of immunostaining for TAMs and IL-6 in CRC tissues from early to late stage. [file 1471-2407-14-330-S1.zip › stage-I-CD-68.TIF]

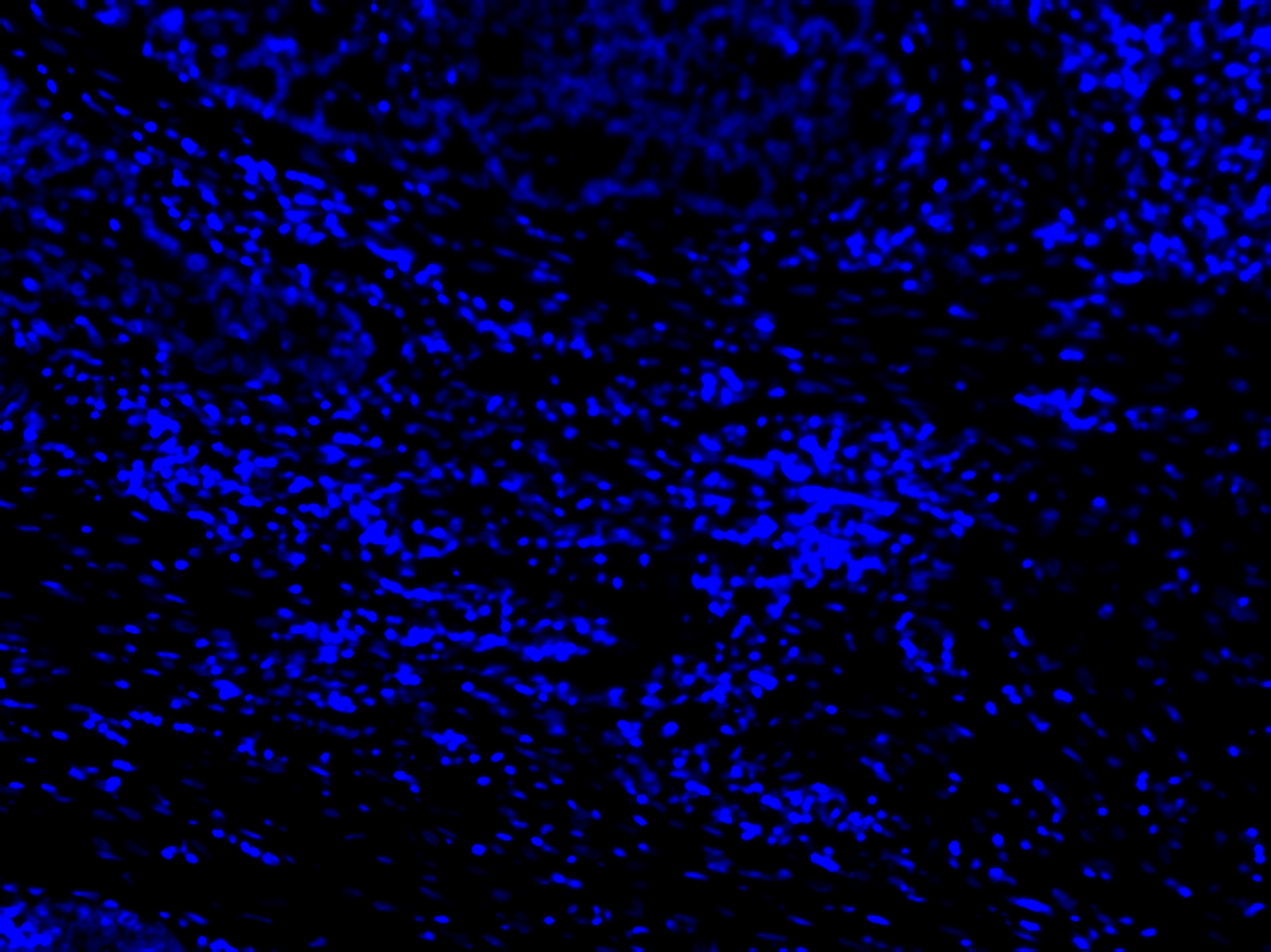

Supplement: Additional file 1 — The original high solution images of immunostaining for TAMs and IL-6 in CRC tissues from early to late stage. [file 1471-2407-14-330-S1.zip › stage-I-DAPI.TIF]

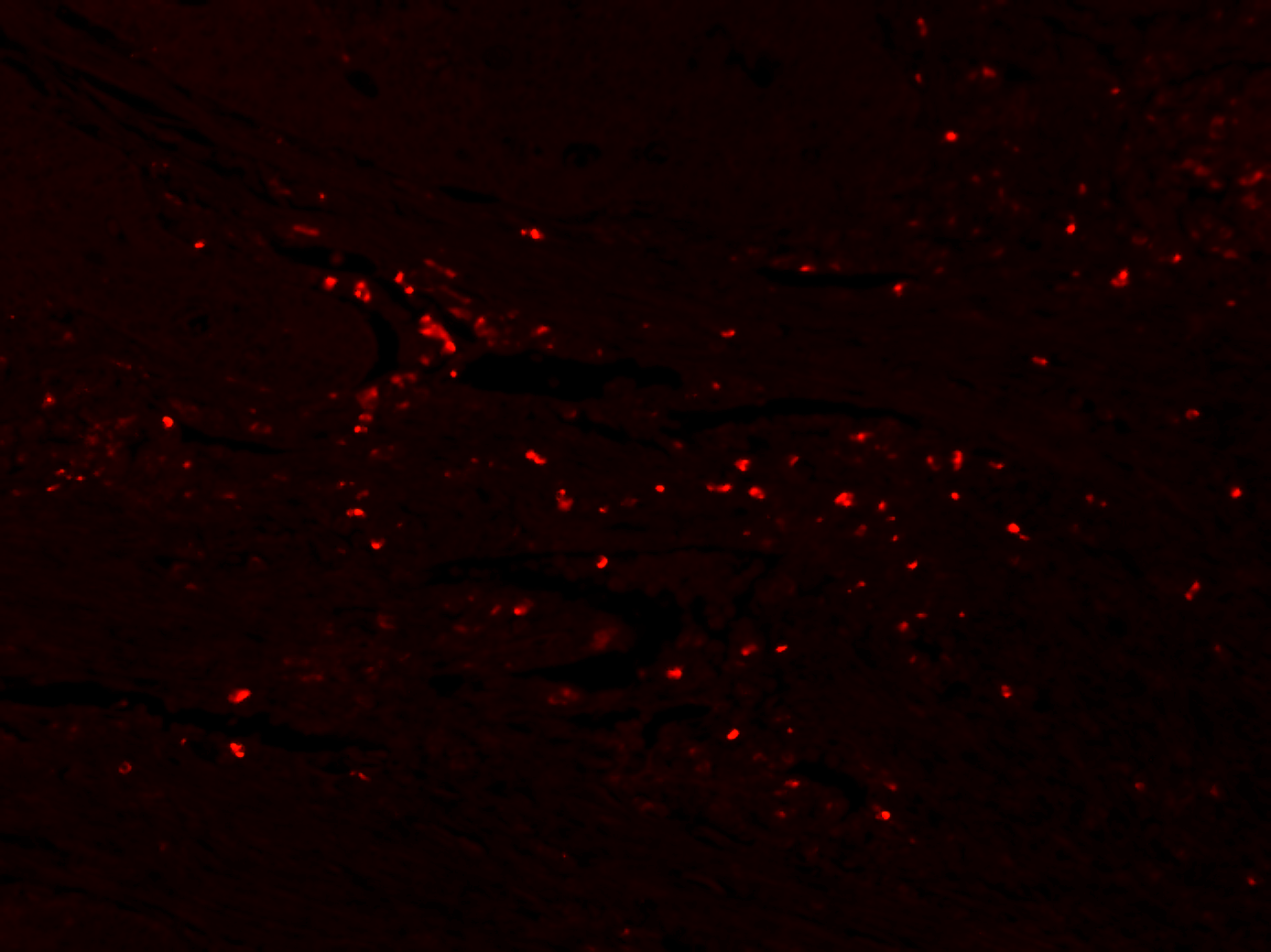

Supplement: Additional file 1 — The original high solution images of immunostaining for TAMs and IL-6 in CRC tissues from early to late stage. [file 1471-2407-14-330-S1.zip › stage-I-IL-6.TIF]

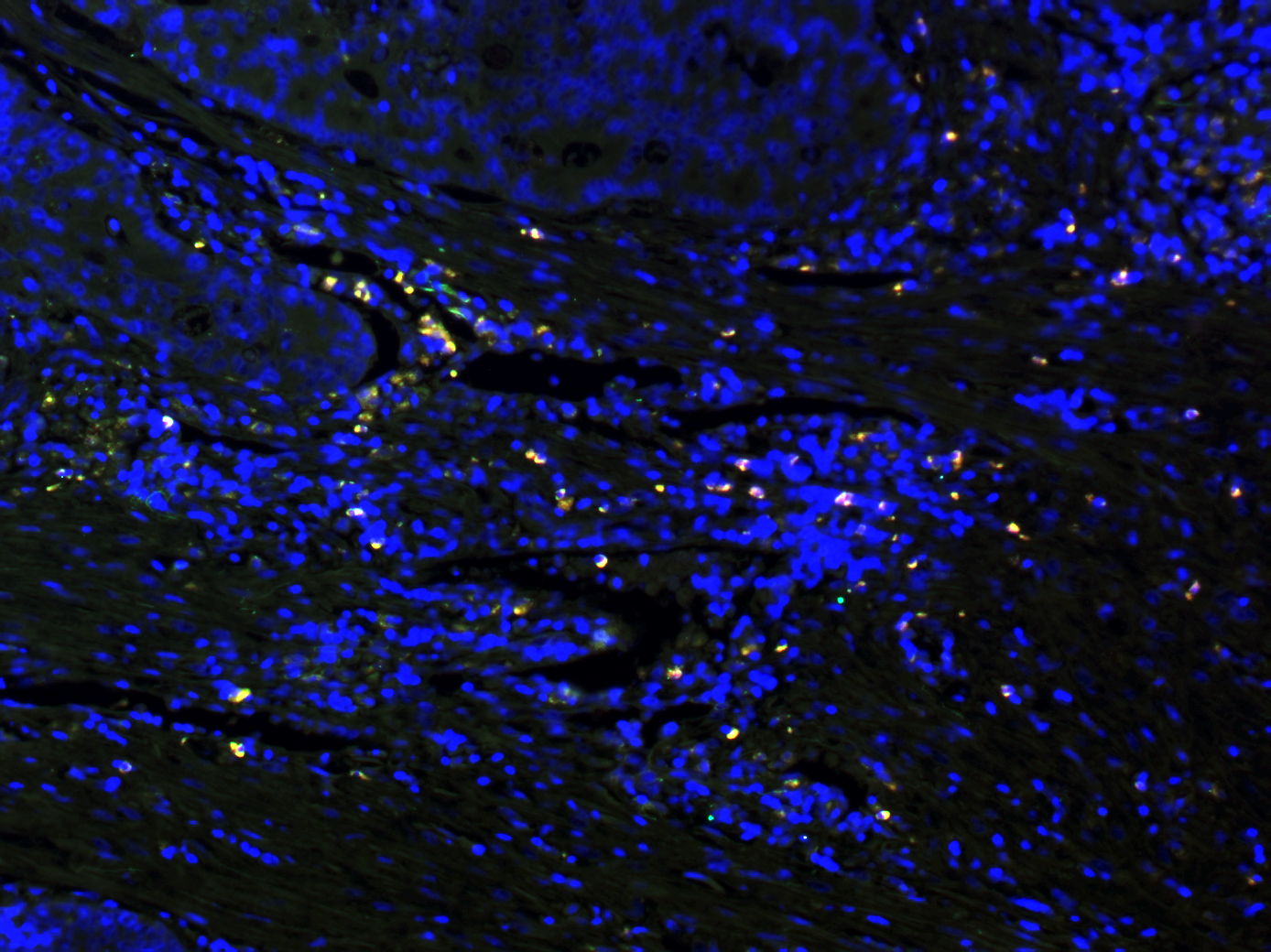

Supplement: Additional file 1 — The original high solution images of immunostaining for TAMs and IL-6 in CRC tissues from early to late stage. [file 1471-2407-14-330-S1.zip › stage-I-IL-6-CD-68-merge.tif]

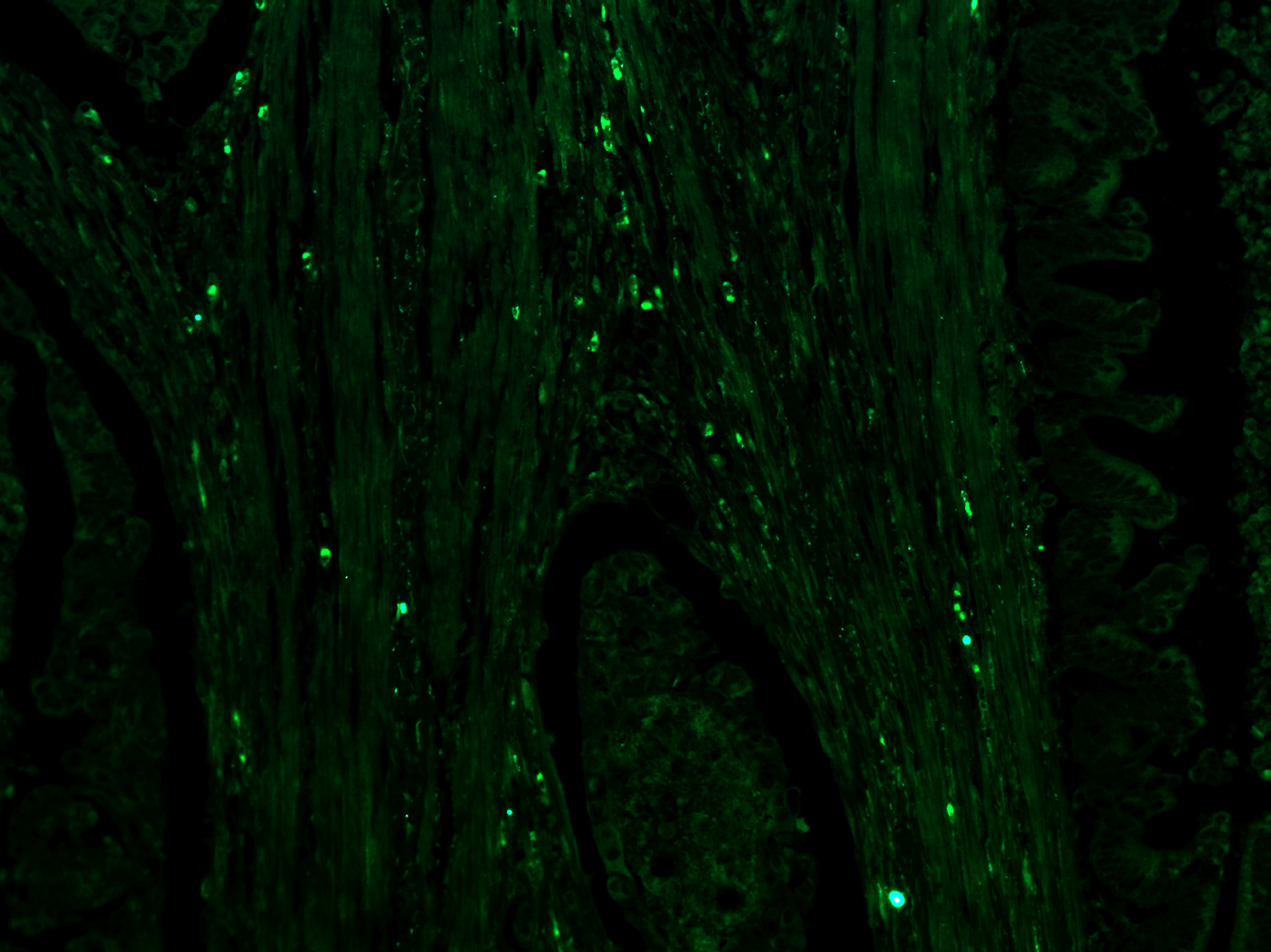

Supplement: Additional file 1 — The original high solution images of immunostaining for TAMs and IL-6 in CRC tissues from early to late stage. [file 1471-2407-14-330-S1.zip › stage-IV-CD-68.TIF]

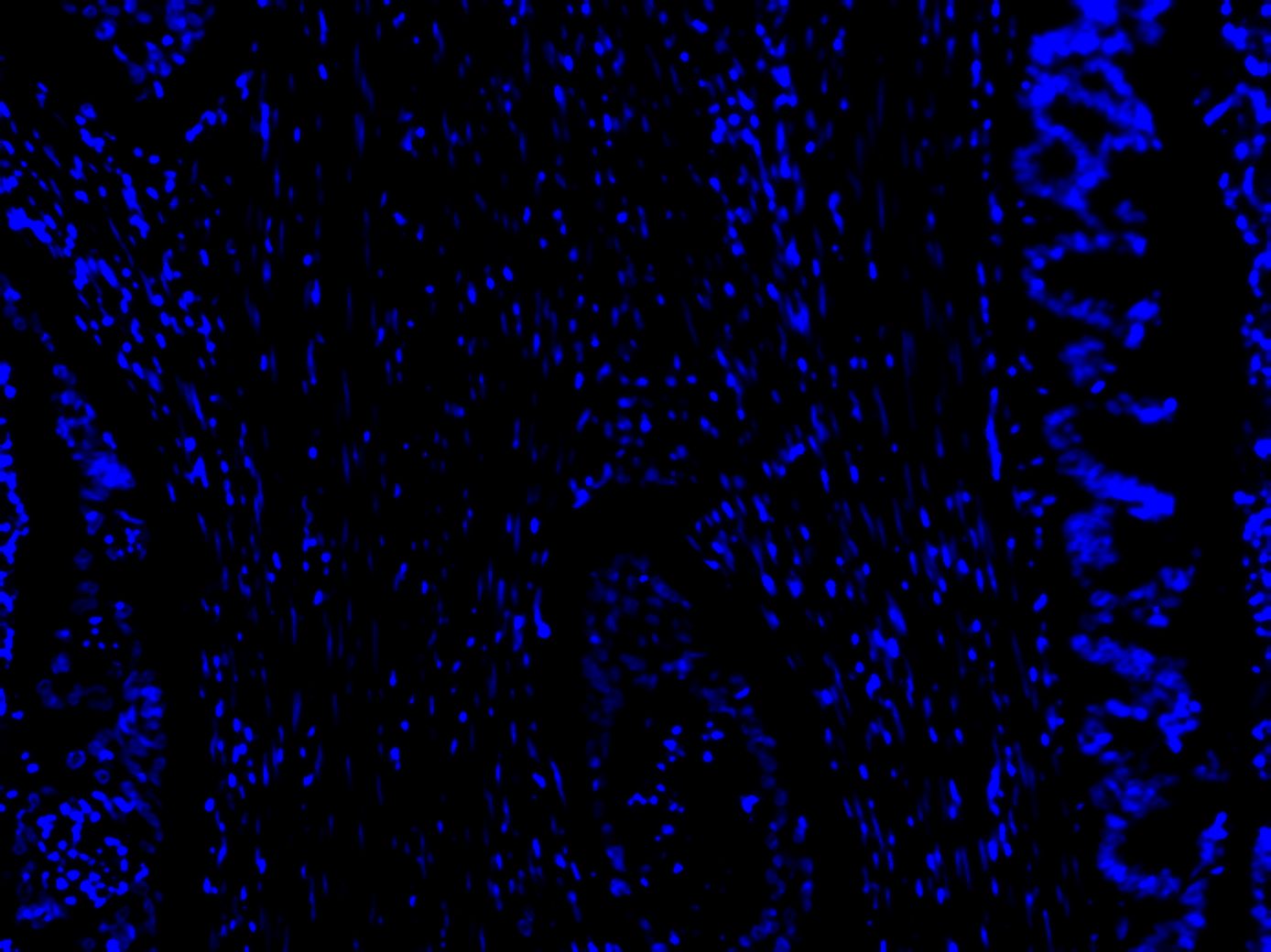

Supplement: Additional file 1 — The original high solution images of immunostaining for TAMs and IL-6 in CRC tissues from early to late stage. [file 1471-2407-14-330-S1.zip › stage-IV-DAPI.TIF]

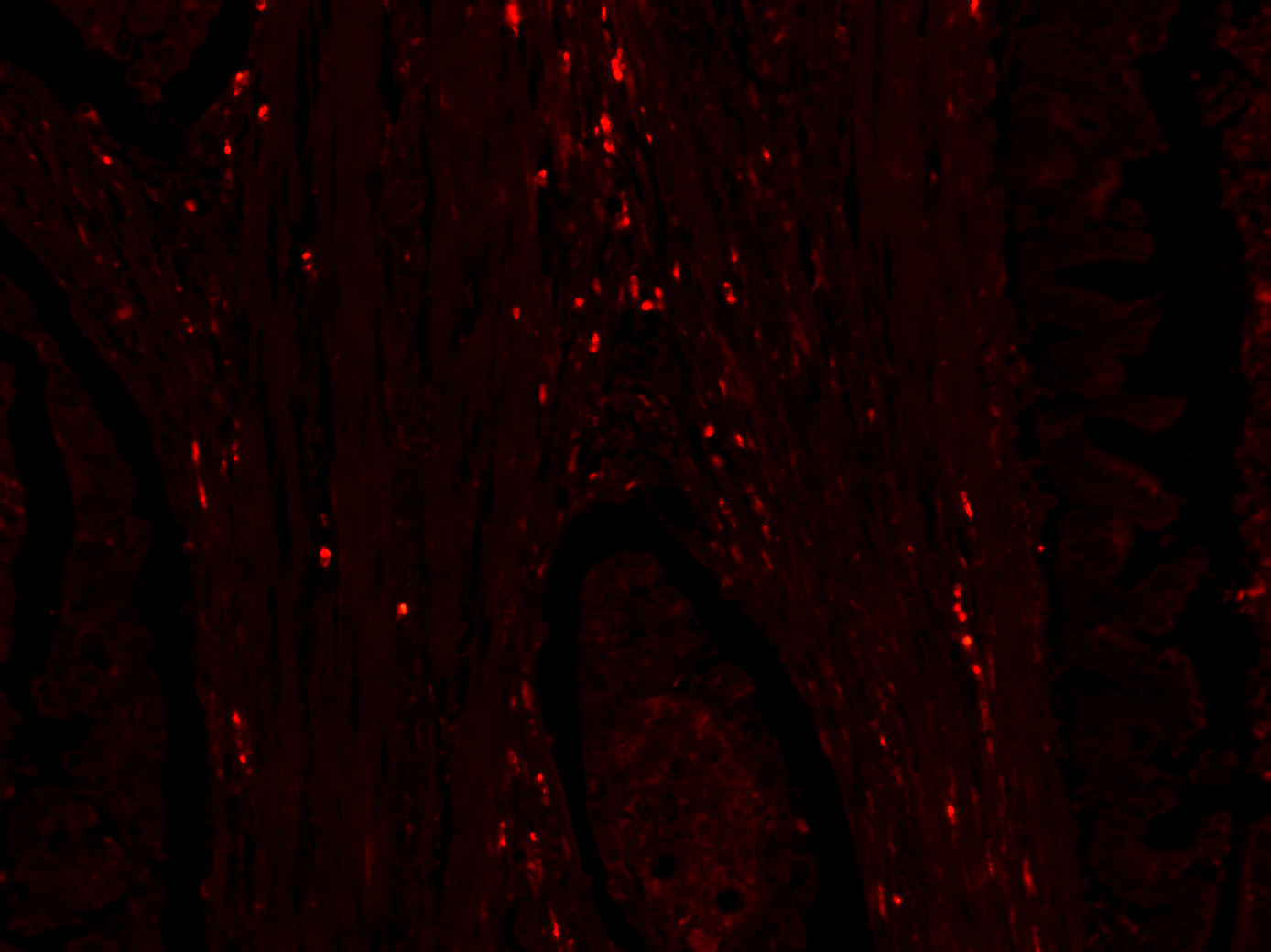

Supplement: Additional file 1 — The original high solution images of immunostaining for TAMs and IL-6 in CRC tissues from early to late stage. [file 1471-2407-14-330-S1.zip › stage-IV-IL-6.TIF]

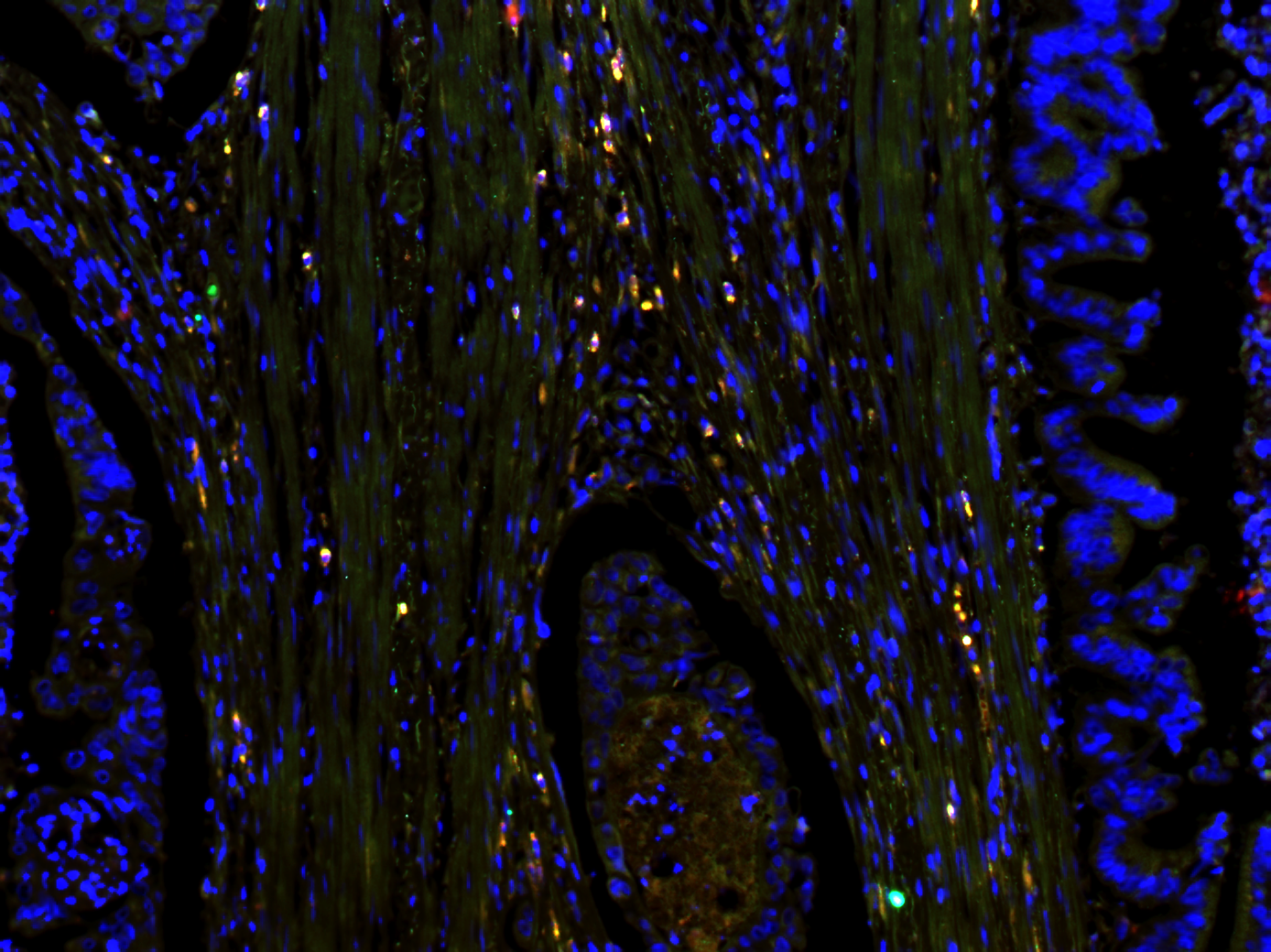

Supplement: Additional file 1 — The original high solution images of immunostaining for TAMs and IL-6 in CRC tissues from early to late stage. [file 1471-2407-14-330-S1.zip › stage-IV-IL-6-CD-68-merge.tif]

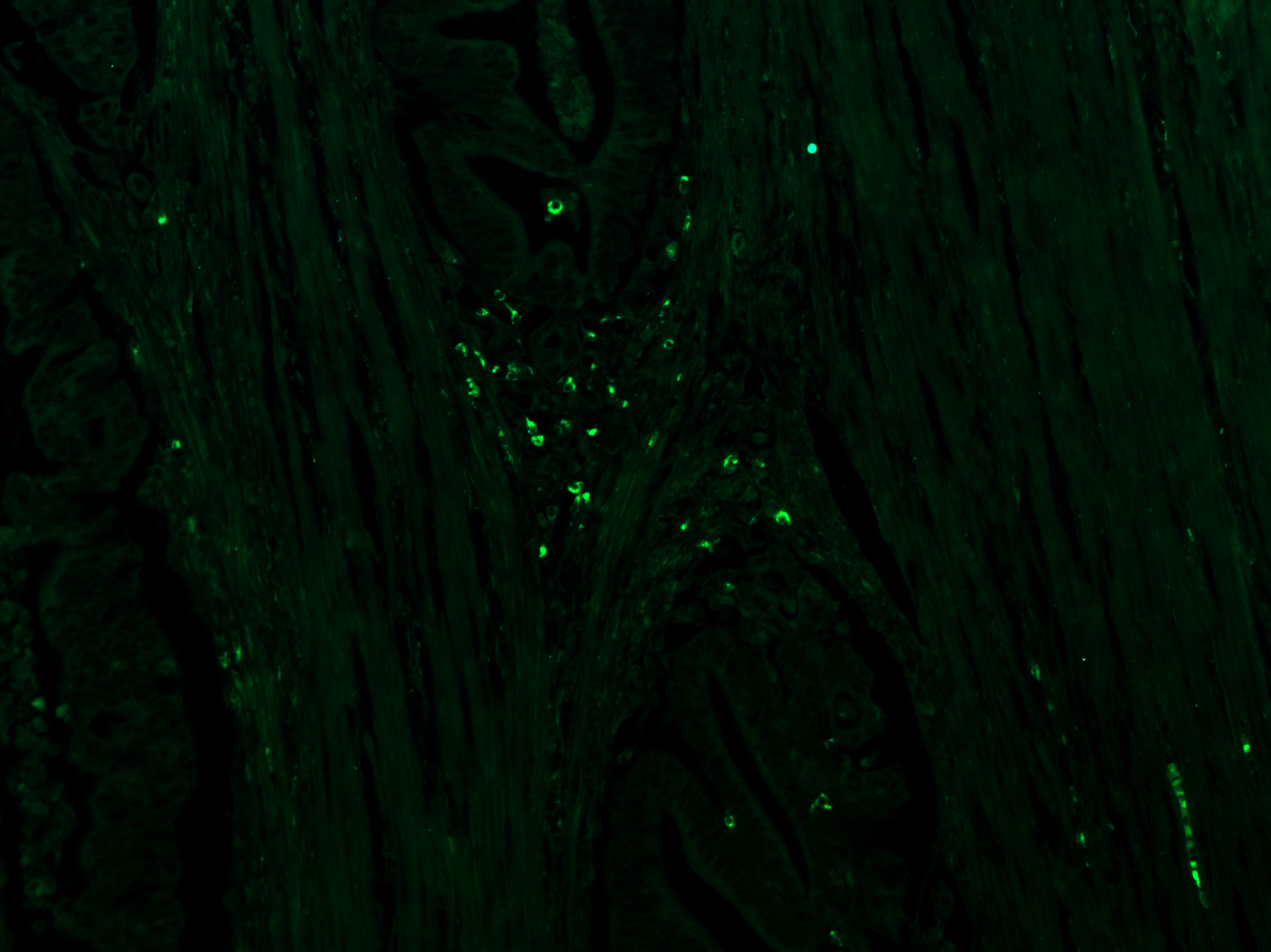

Supplement: Additional file 2 — The original high solution images of immunostaining for TAMs and IL-8 in CRC tissues from early to late stage. [file 1471-2407-14-330-S2.zip › stage-I-CD-68.TIF]

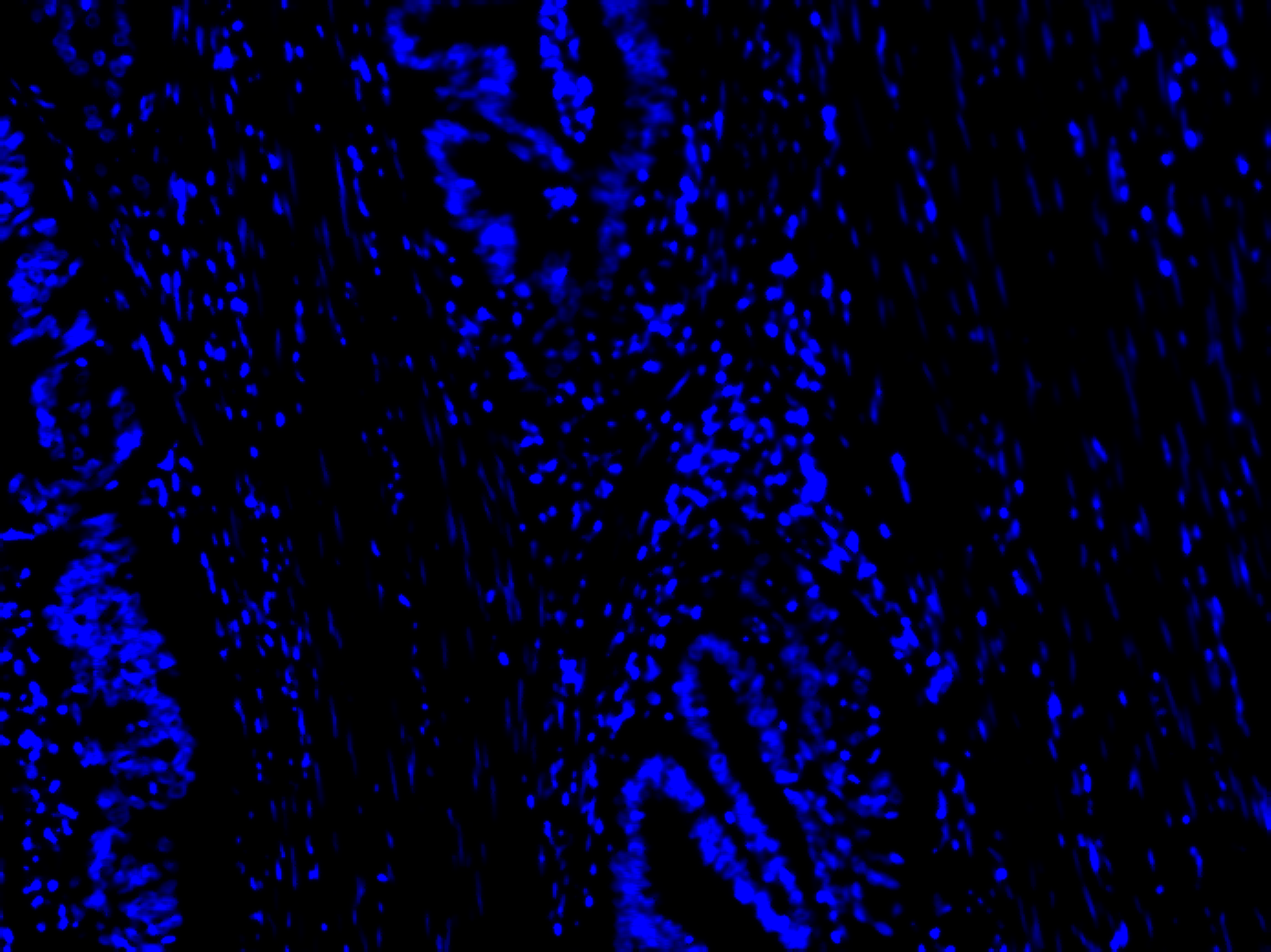

Supplement: Additional file 2 — The original high solution images of immunostaining for TAMs and IL-8 in CRC tissues from early to late stage. [file 1471-2407-14-330-S2.zip › stage-I-DAPI.TIF]

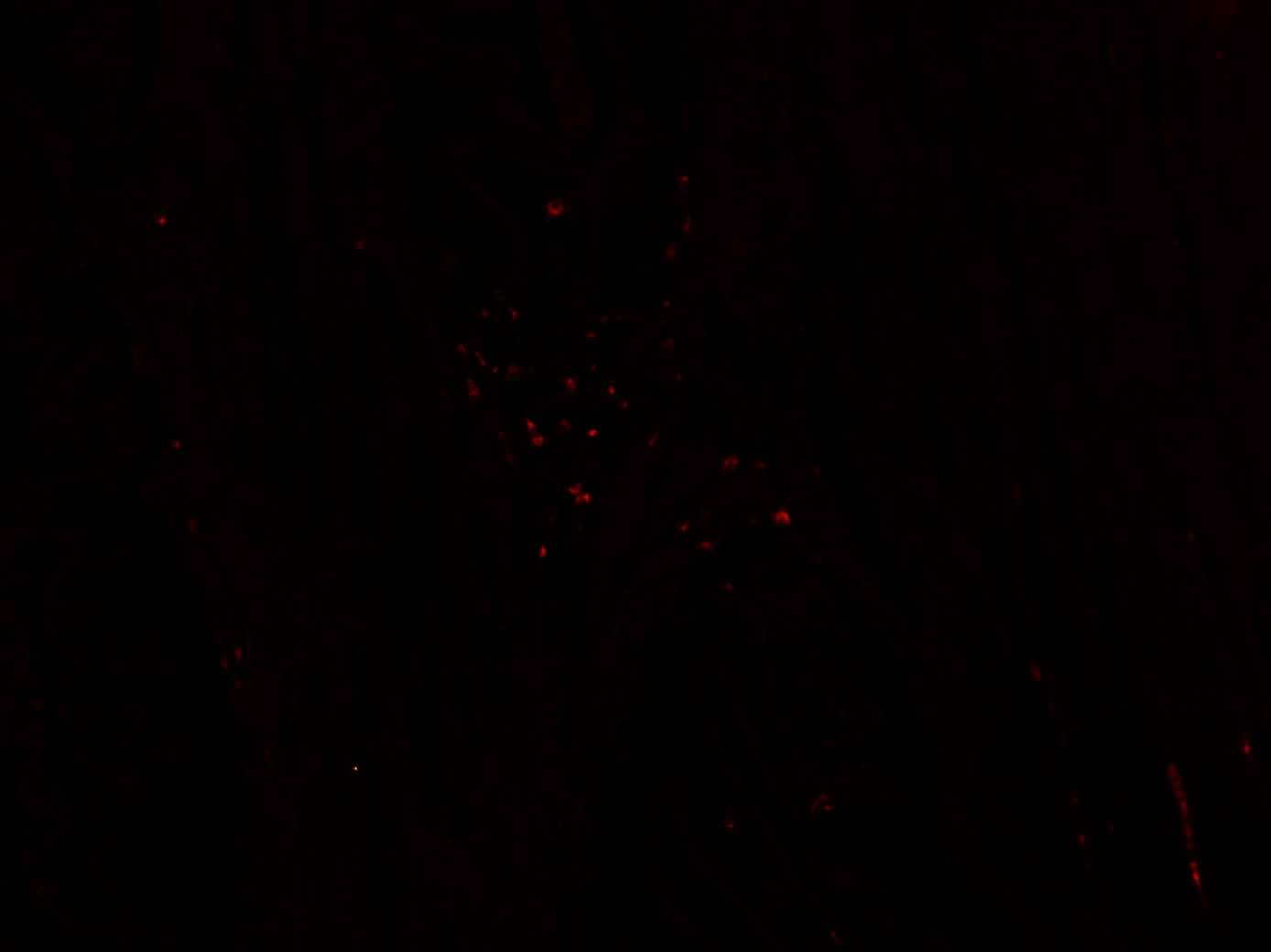

Supplement: Additional file 2 — The original high solution images of immunostaining for TAMs and IL-8 in CRC tissues from early to late stage. [file 1471-2407-14-330-S2.zip › stage-I-IL-8.TIF]

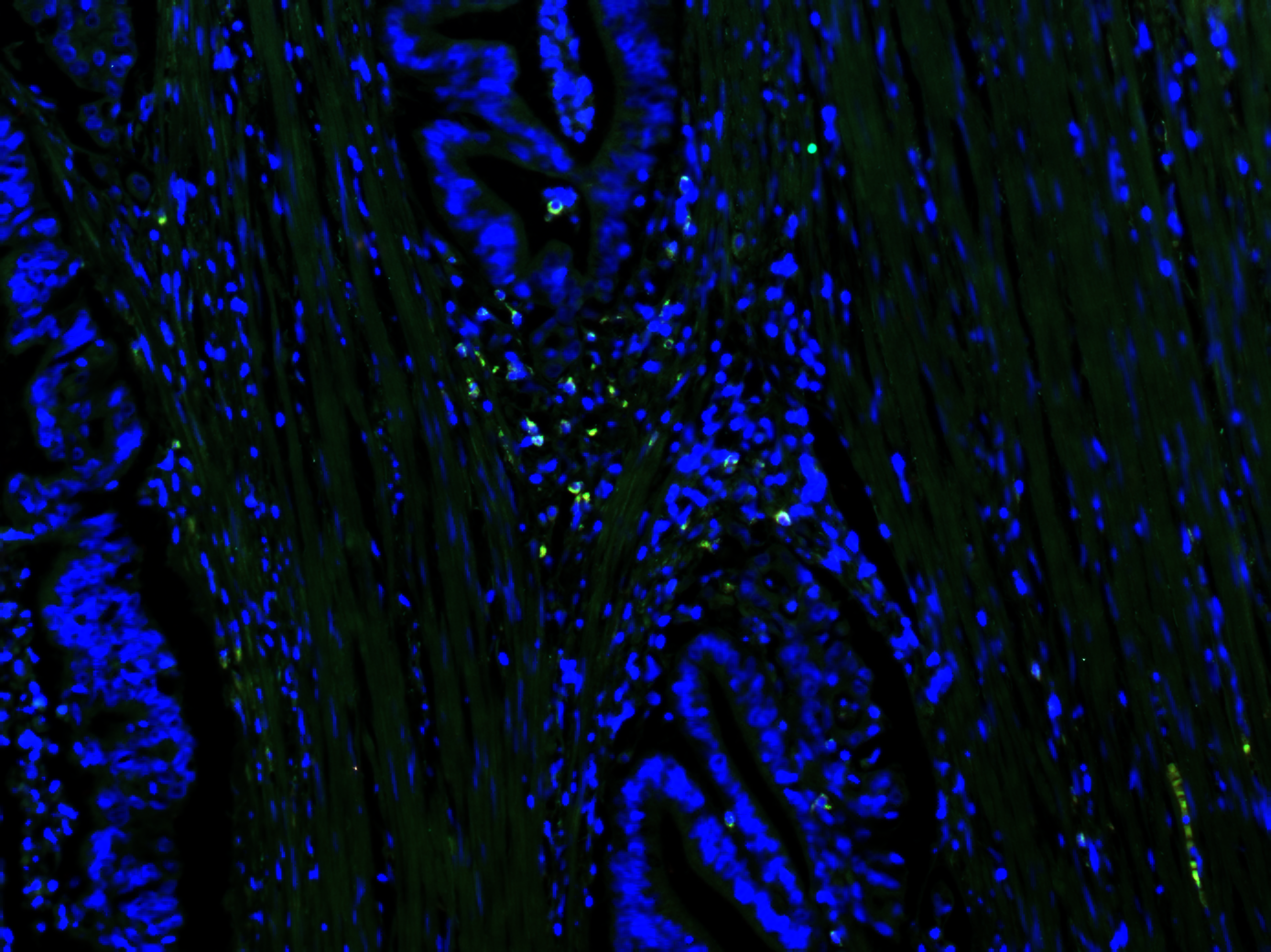

Supplement: Additional file 2 — The original high solution images of immunostaining for TAMs and IL-8 in CRC tissues from early to late stage. [file 1471-2407-14-330-S2.zip › stage-I-IL-8-CD-68-merge.tif]

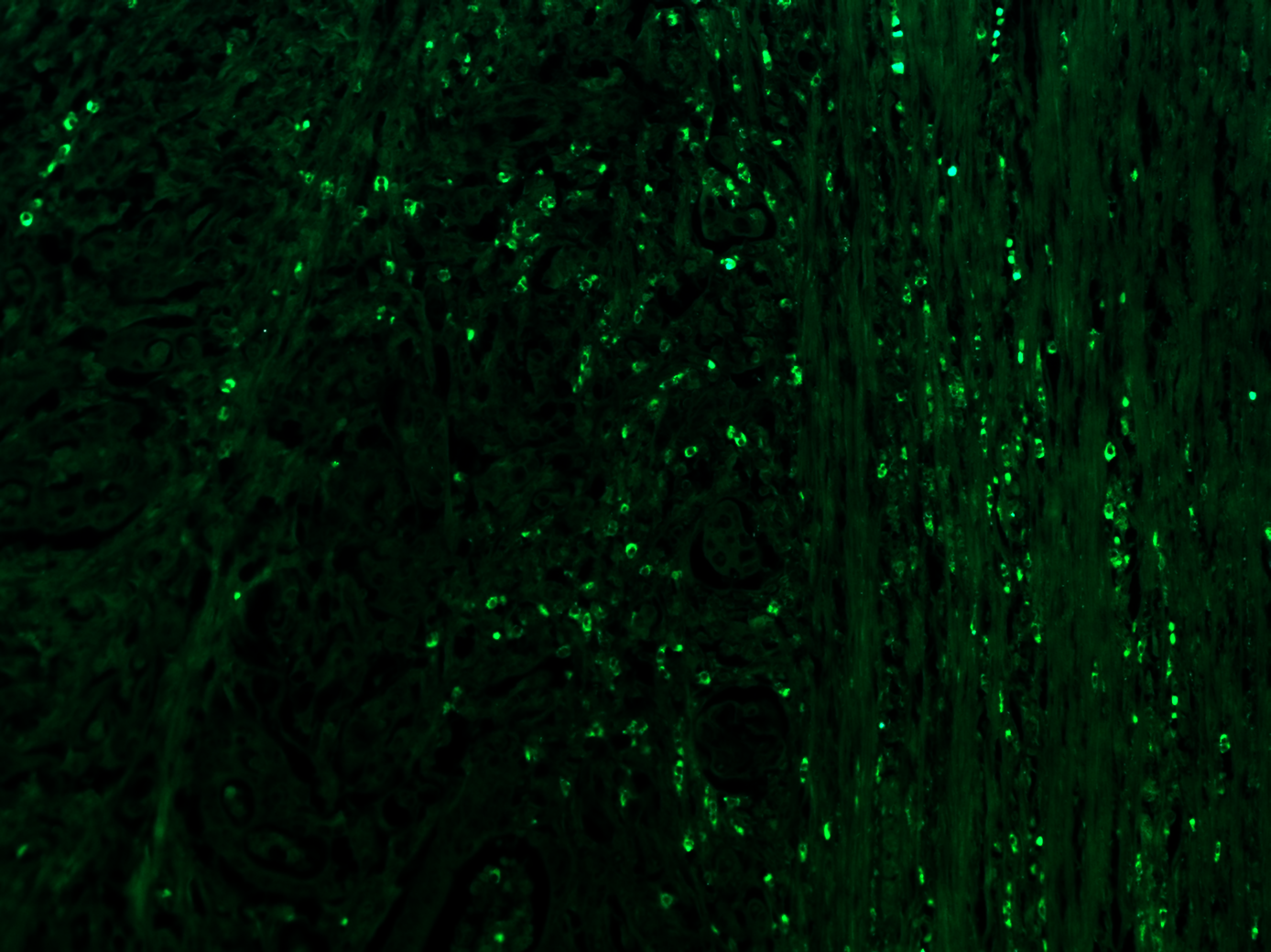

Supplement: Additional file 2 — The original high solution images of immunostaining for TAMs and IL-8 in CRC tissues from early to late stage. [file 1471-2407-14-330-S2.zip › stage-IV-CD-68.TIF]

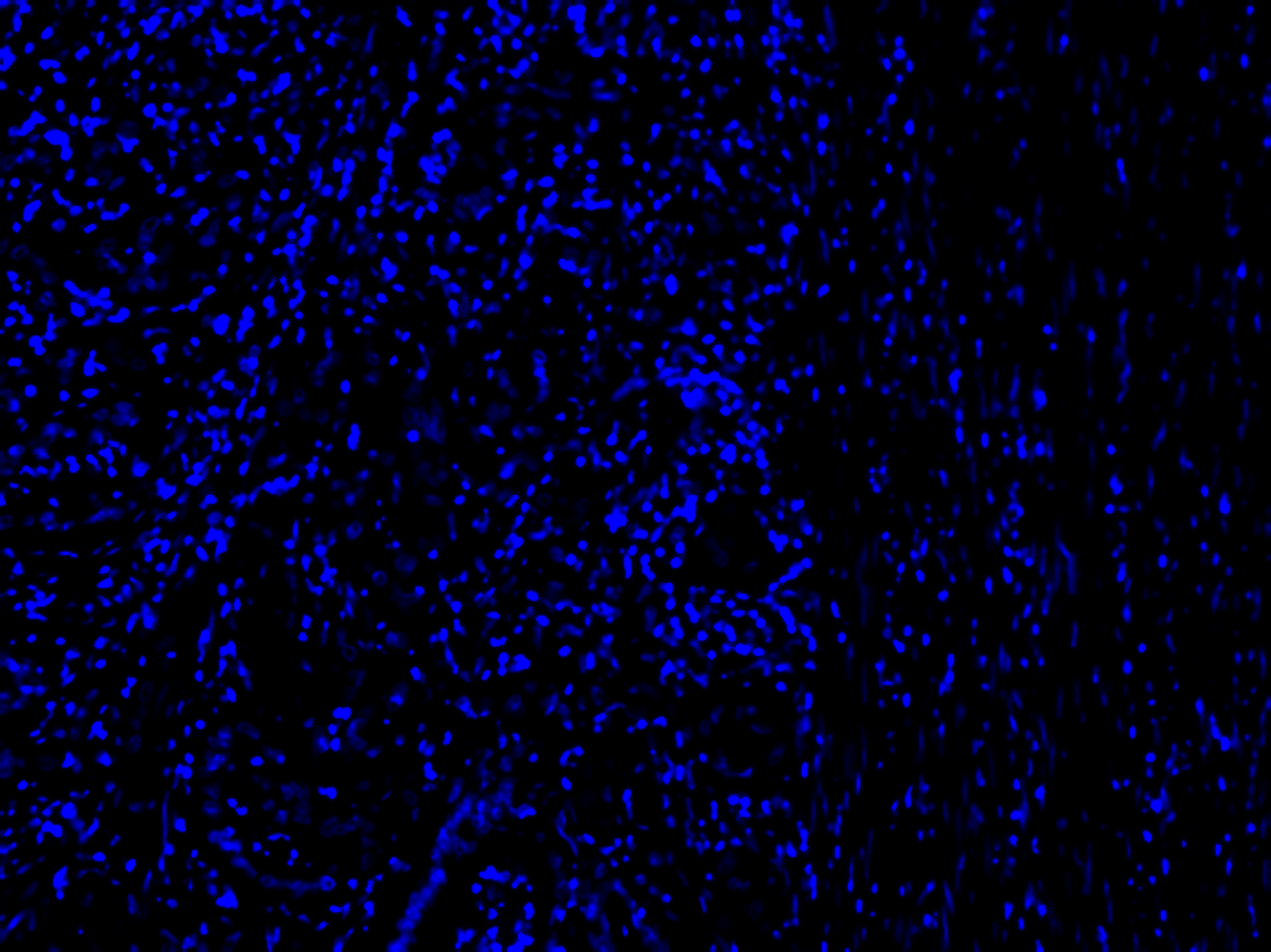

Supplement: Additional file 2 — The original high solution images of immunostaining for TAMs and IL-8 in CRC tissues from early to late stage. [file 1471-2407-14-330-S2.zip › stage-IV-DAPI.TIF]

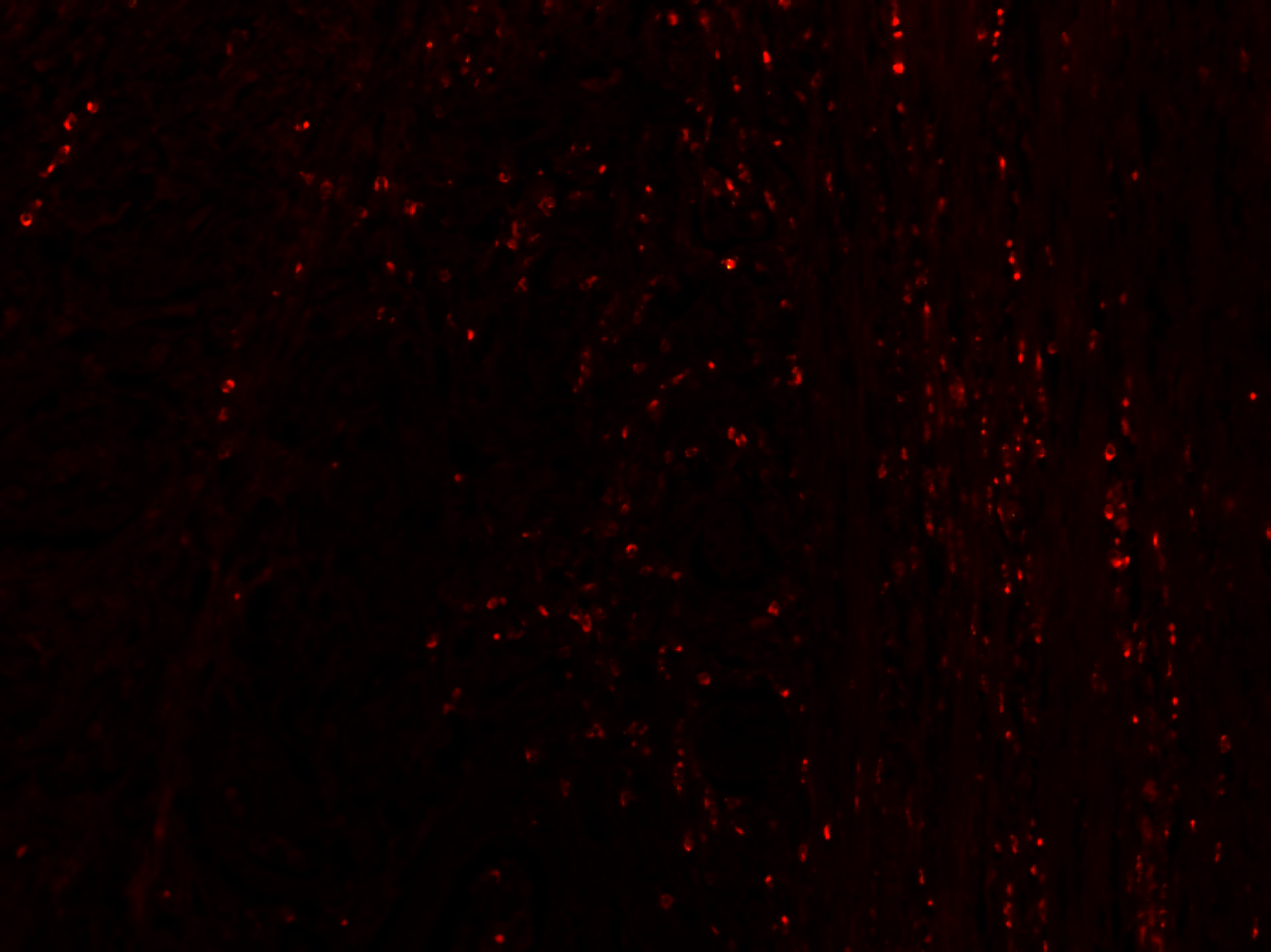

Supplement: Additional file 2 — The original high solution images of immunostaining for TAMs and IL-8 in CRC tissues from early to late stage. [file 1471-2407-14-330-S2.zip › stage-IV-IL-8.TIF]

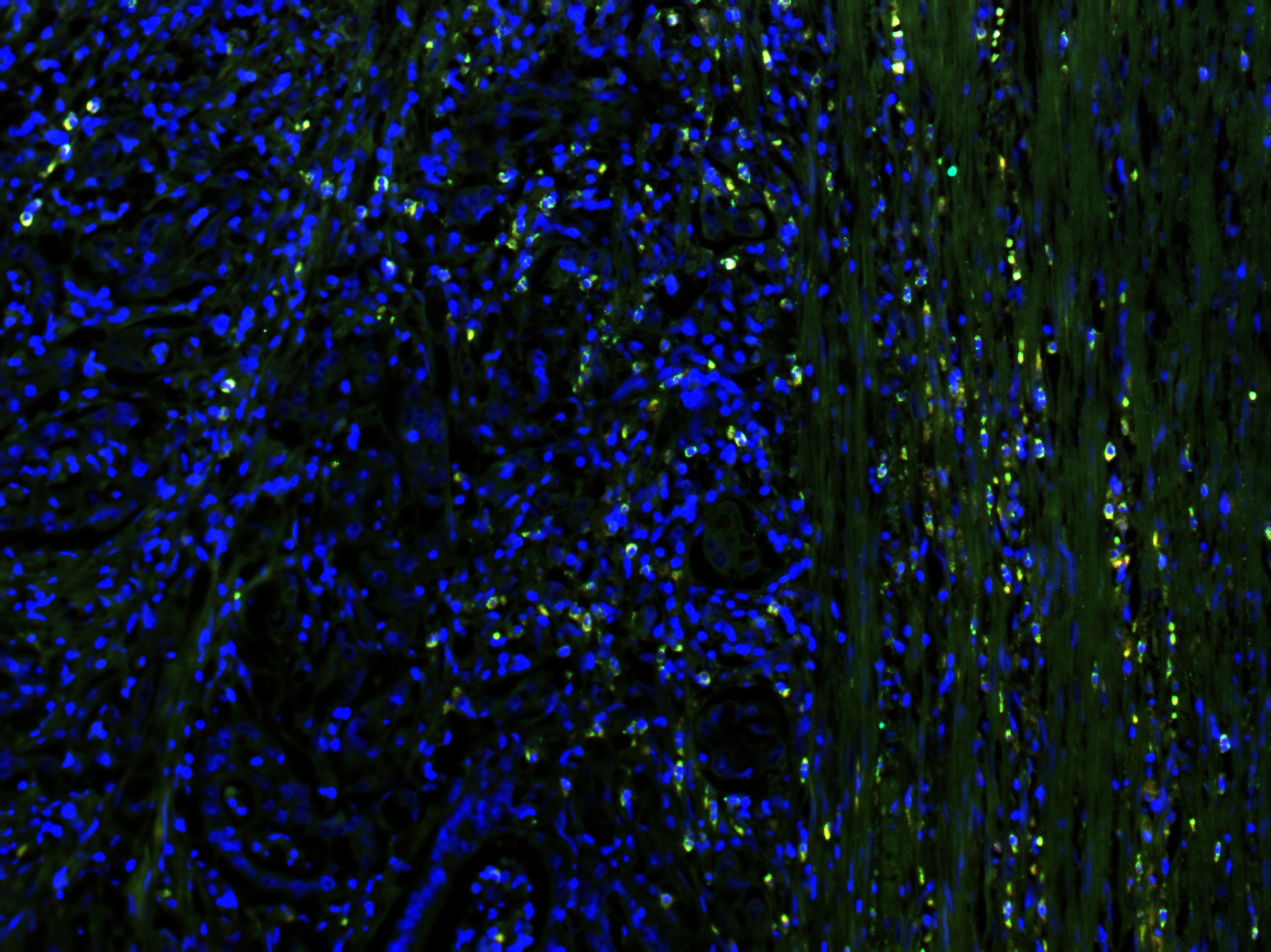

Supplement: Additional file 2 — The original high solution images of immunostaining for TAMs and IL-8 in CRC tissues from early to late stage. [file 1471-2407-14-330-S2.zip › stage-IV-IL-8-CD-68-merge.tif]
